# Graphene quantum dots in alveolar macrophage: uptake-exocytosis, accumulation in nuclei, nuclear responses and DNA cleavage

**DOI:** 10.1186/s12989-018-0279-8

**Published:** 2018-11-13

**Authors:** Lina Xu, Yanhui Dai, Zhenyu Wang, Jian Zhao, Fei Li, Jason C. White, Baoshan Xing

**Affiliations:** 1 Institute of Environmental Processes and Pollution Control, and School of Environmental and Civil Engineering, Jiangnan University, Wuxi, 214122 China; 2 Institute of Coastal Environmental Pollution Control, and Ministry of Education Key Laboratory of Marine Environment and Ecology, Ocean University of China, Qingdao, 266100 China; 3 Laboratory for Marine Ecology and Environmental Science, Qingdao National Laboratory for Marine Science and Technology, Qingdao, 266071 China; 4 Key Laboratory of Coastal Zone Environmental Processes and Ecological Remediation, Yantai Institute of Coastal Zone Research (YIC), Chinese Academy of Sciences (CAS), Yantai, 264003 China; 5 Department of Analytical Chemistry, The Connecticut Agricultural Experiment Station, New Haven, CT 06504 USA; 6 Stockbridge School of Agriculture, University of Massachusetts, Amherst, MA 01003 USA

**Keywords:** Aminated graphene quantum dots, Macrophages, Endocytosis, Nuclear accumulation, DNA cleavage, Molecular docking

## Abstract

**Background:**

Given the tremendous potential for graphene quantum dots (QDs) in biomedical applications, a thorough understanding of the interaction of these materials with macrophages is essential because macrophages are one of the most important barriers against exogenous particles. Although the cytotoxicity and cellular uptake of graphene QDs were reported in previous studies, the interaction between nuclei and the internalized graphene QDs is not well understood. We thus systematically studied the nuclear uptake and related nuclear response associated with aminated graphene QDs (AG-QDs) exposure.

**Results:**

AG-QDs showed modest 24-h inhibition to rat alveolar macrophages (NR8383), with a minimum inhibitory concentration (MIC) of 200 μg/mL. Early apoptosis was significantly increased by AG-QDs (100 and 200 μg/mL) exposure and played a major role in cell death. The internalization of AG-QDs was mainly via energy-dependent endocytosis, phagocytosis and caveolae-mediated endocytosis. After a 48-h clearance period, more than half of the internalized AG-QDs remained in the cellular cytoplasm and nucleus. Moreover, AG-QDs were effectively accumulated in nucleus and were likely regulated by two nuclear pore complexes genes (*Kapβ2* and *Nup98*). AG-QDs were shown to alter the morphology, area, viability and nuclear components of exposed cells. Significant cleavage and cross-linking of DNA chains after AG-QDs exposure were confirmed by atomic force microscopy investigation. Molecular docking simulations showed that H-bonding and π-π stacking were the dominant forces mediating the interactions between AG-QDs and DNA, and were the important mechanisms resulting in DNA chain cleavage. In addition, the generation of reactive oxygen species (ROS) (e.g.*,* •OH), and the up-regulation of caspase genes also contributed to DNA cleavage.

**Conclusions:**

AG-QDs were internalized by macrophages and accumulated in nuclei, which further resulted in nuclear damage and DNA cleavage. It is demonstrated that oxidative damage, direct contact via H-bonding and π-π stacking, and the up-regulation of caspase genes are the primary mechanisms for the observed DNA cleavage by AG-QDs.

**Electronic supplementary material:**

The online version of this article (10.1186/s12989-018-0279-8) contains supplementary material, which is available to authorized users.

## Background

Graphene quantum dots (QDs) are a zero-dimensional graphitic nanomaterial with exceptional physical and chemical properties and have inspired significant research efforts since being firstly developed [1, 2]. Owing to their stable photoluminescence, chemical inertness, and compatible functionalization with biomolecules, graphene QDs have been evaluated for their potential use in biomedical applications such as bioimaging, biosensing, and drug/gene delivery [3–5]. The cellular fate and potential toxicity of graphene QDs are critical issues facing successful biomedical research and application [6].

Although current investigations with graphene QDs have suggested that these materials have low toxicity to mammalian cells, focused study on the detailed interaction of these materials with cellular systems and organelles need to be conducted. It has been shown that graphene QDs can be internalized by human cells. Wu et al. found that caveolae-mediated endocytosis was the major pathway for graphene QDs uptake by human MGC-803 cells [7]. Wang et al. reported that direct penetration rather than energy-dependent pathways (e.g., endocytosis) was primarily responsible for graphene QDs uptake by human fibroblast cells [8]. Although most of studies have shown that graphene QDs randomly distribute in cytoplasm and do not diffuse into the nucleus of mammalian cells such as A549 and osteoblastic cells (MC3T3-E1) [9, 10], Wu et al. did report that graphene QDs entered the nucleus of MCF-7 cells [7]. These apparent contradictory findings clearly highlight the need for additional study on the pathways for cellular and nuclear uptake of graphene QDs. To the best of our knowledge, no report on the internalization of graphene QDs within mammalian macrophages is observed, although there are two studies focusing on cytotoxicity to Thp-1 macrophages [11, 12]. Macrophages are one of most important barriers against exogenous particles/agents, and are the dominant infiltrating cells rapidly responding to biomaterial implantation in biomedical applications [13]. Therefore, we comprehensively investigated the translocation of graphene QDs in macrophages, including uptake pathways, exocytosis, and cellular/nuclear distribution.

It has been shown that DNA in NIH-3 T3 cells could be damaged via oxidative stress upon exposure to graphene QDs without the direct contact with nuclear genetic materials [8]. This DNA damage is expected to be stronger if graphene QDs were to enter the nucleus and directly contact with DNA, although changes in apparent toxicity (e.g., growth inhibition) may not be obvious. It is shown that graphene QDs could be intercalated in the DNA base pairs during direct incubation [14]. Another study found that graphene QDs interacted more strongly with DNA than did graphene oxide (GO) [15]. π–π Stacking and hydrogen bonding are likely the dominant forces that overcome electrostatic repulsion as shown for the interaction of DNA with micrometer-sized GO and reduced GO (rGO) [16, 17]. However, the behavior of graphene QDs in nucleus and underlying mechanism for the interaction of these particles with DNA remain unknown. We hypothesize that graphene QDs could damage DNA during direct contact/binding after nuclear uptake, which could further lead to the abnormal responses of nuclei and genetic material.

In the current study, the nuclear uptake, DNA damage and related cellular responses after graphene QD exposure to rat alveolar macrophages (NR8383) were investigated. Amine-modified graphene QDs (AG-QDs) were used because amine groups significantly promote linkage between graphene-materials and DNA [18]. Based on the two hypotheses above, this study specifically investigated (1) the uptake, distribution and nuclear internalization of AG-QDs in macrophages; and (2) the interaction of AG-QDs with DNA through atomic force microscopic analysis and molecular simulation. The findings from this work will provide new insights into the detailed behavior of graphene QDs in cells and nuclei, and will be useful for better understanding the biosafety of these novel graphene materials.

## Results

### AG-QDs characterization and impact on cell viability

**Fig. 1:**
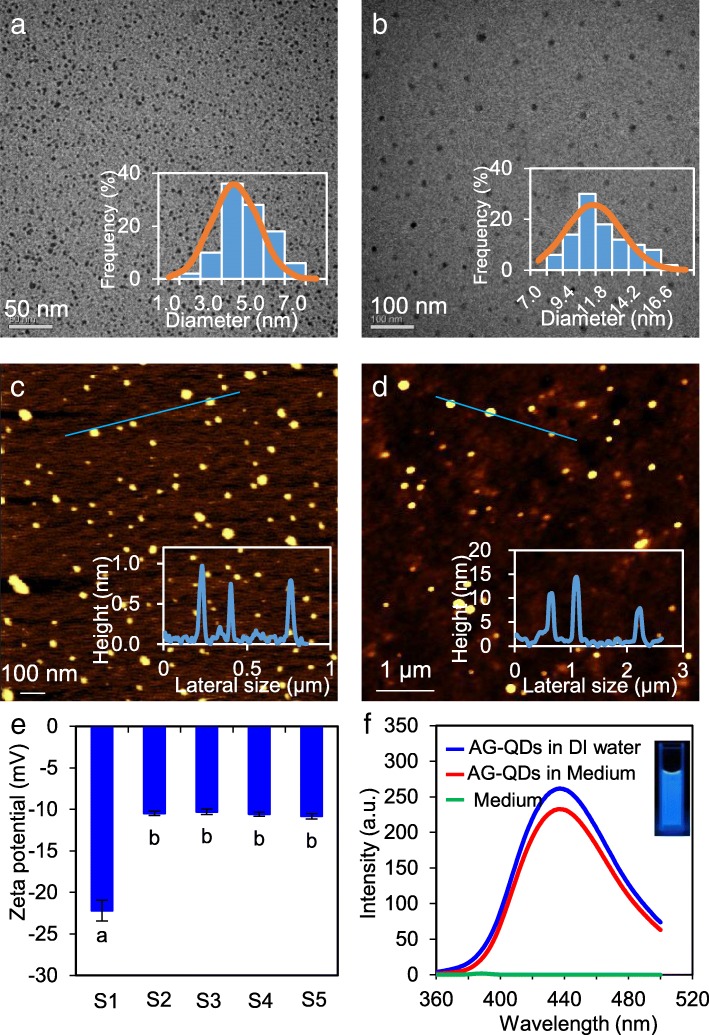
Characterization of AG-QDs. **a**, **b**: TEM imaging and size distribution of AG-QDs in deionized (DI) water and culture medium, respectively. **c**, **d**: AFM topography and height of AG-QDs in DI water and culture medium, respectively. **e** Zeta potentials of AG-QDs in DI water and culture medium. In panel (**e**), S1 represents AG-QDs (50 μg/mL) in DI water, and S2-S5 represent AG-QDs at 0, 50, 100, and 200 μg/mL in FBS-containing culture medium, respectively. (**f**) Fluorescence spectra of AG-QDs in DI water and culture medium. In panels (**a**) and (**b**), the size distribution of AG-QDs (as shown by the inserted figures) was obtained by calculating 50 randomly-selected points during TEM imaging. In panels (**c**) and (**d**), the inserted figures were the height profiles of AG-QDs as marked on the AFM images. In panel (**e**), the values are given as mean ± SD (standard deviation). Significant difference is marked with different letters (*p* < 0.05, LSD, *n* = 6)

Elemental analysis by X-ray photoelectron spectroscopy (XPS) (Additional file 1: Figure S1a) showed that the O/C atomic ratio for AG-QDs was 0.525, demonstrating that the particles had a relatively high oxidation level. The N/C atomic ratio was calculated to be 0.140, which is similar to nitrogen-doped graphene QDs (N/C = 0.178) [19]. The characteristic peaks of C 1 s at 284.8, 285.9, 286.6, 288.4 and 289.0 eV represented C=C/C-C (43.6%), C-N (11.8%) C-OH (12.3%), C=O (13.6%) and O=C-OH (18.7%) groups, respectively (Additional file 1: Figure S1b). In addition, N 1 s XPS spectra showed that the C-N groups on the surface of AG-QDs were mainly N-H (399.6 eV, 52.8%), -N^+^ = (400.9 eV, 38.1%) and -N=C (398.5 eV, 9.10%) (Additional file 1: Figure S1c), clearly highlighting the dominance of -NH_2_ functional groups. With regard to the morphology of AG-QDs in DI water, transmission electron microscopy (TEM) images show that individual AG-QDs are uniform, with an average particle size of ~ 4.1 nm (Fig. 1a). AG-QDs thickness was calculated as ~ 0.720 nm (Fig. 1c), corresponding to a single layer of oxidized graphene [20]. After incubation in the culture medium for 24 h, the size and thickness of individual AG-QDs were 9.40–11.8 nm and 4.30–10.2 nm, respectively (Fig. 1b, d), suggesting the adsorption of medium components (e.g., fetal bovine serum (FBS)) onto the particles. AG-QDs (50 μg/mL) were negatively charged (− 22.2 mV) in DI water (Fig. 1e) due to the presence of more carboxyl groups than amino groups on surface (Additional file 1: Figure S1b). The zeta potentials of AG-QDs at different concentrations (50, 100, 200 μg/mL) in cell culture medium were less negative (− 10.3~ − 10.8 mV) than in DI water (Fig. 1e) because of the adsorption of FBS (− 10.5 mV) on the particle surface [21]. Hydrodynamic diameter of AG-QDs (50 μg/mL) in DI water was 29.7 nm (Additional file 1: Figure S2), suggesting the formation of AG-QDs homoaggregates. In the culture medium, the hydrodynamic diameter of AG-QDs was much larger (113.8 nm), which may be caused by the adsorption of FBS and formation of protein corona. In addition, the AG-QDs retained a significant characteristic blue fluorescence at 438 nm after 96-h incubation in the cell culture medium (Fig. 1f).

Cell viability of the rat alveolar macrophages (NR8383) was examined after AG-QDs exposure using the CCK-8 assay. After 24-h exposure, no significant inhibition on cell growth was observed after particle exposure at 10, 25, 50 and 100 μg/mL; however, particle exposure at 200 and 500 μg/mL significantly reduced cell growth (*p* < 0.05) (Additional file 1: Figure S3). After exposure for 48, 72, and 96 h, the minimum inhibitory concentrations (MICs) of AG-QDs were 100, 25, and 25 μg/mL, respectively. These results demonstrate that AG-QDs toxicity is both time- and concentration-dependent. Mouse osteoblastic cells (MC3T3-E1) which play a crucial role in bone formation [22] were also used to assess the toxicity of AG-QDs. The MICs of AG-QDs to MC3T3-E1 cells were 50 μg/mL after exposure for 24–96 h (Additional file 1: Figure S4), confirming the low cytotoxicity of AG-QDs to normal mammalian cells. We subsequently selected AG-QDs exposure concentrations at or lower than the MIC to further investigate cellular uptake of AG-QDs by macrophages and biological response to particle exposure in the following experiments.

**Fig. 2:**
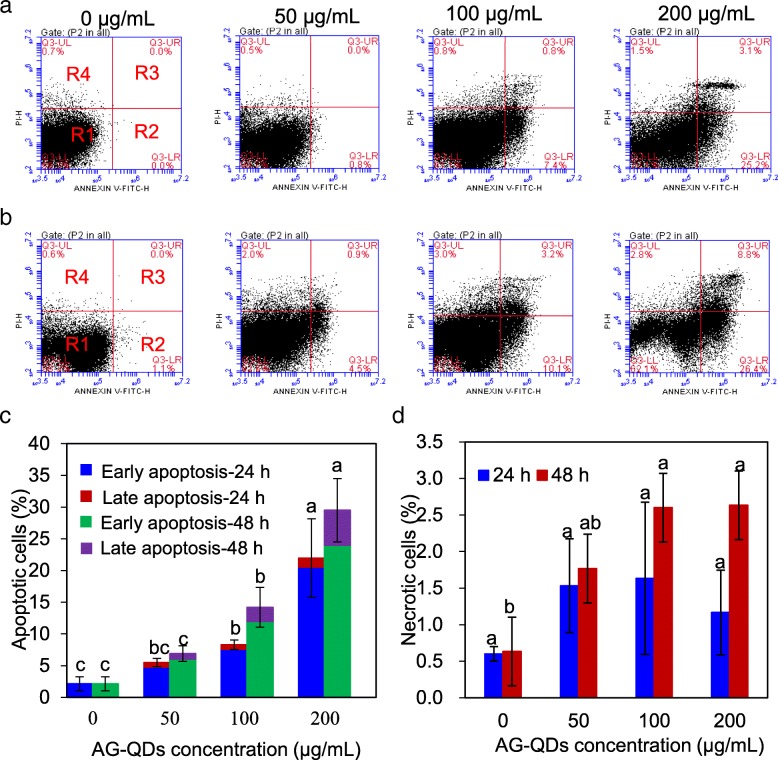
Apoptosis and necrosis of NR8383 cells during 24- and 48-h AG-QDs exposure as detected by flow cytometry. **a**, **b**: Representative flow cytometer images of NR8383 cells after exposed to AG-QDs (0, 50, 100 and 200 μg/mL) for 24, and 48 h. For each image in panels (**a**) and (**b**), the areas R1, R2, R3, and R4 indicate viability, early apoptosis, late apoptosis, and necrotic cells, respectively. **c**, **d**: Quantitative results of apoptotic and necrotic percentages from flow cytometry analysis. In panels (**c**) and (**d**), for a given exposure time, significant difference on apoptotic or necrotic cells among AG-QDs concentrations is marked with different letters (*p* < 0.05, LSD test, *n* = 6)

Macrophage apoptosis and necrosis were further examined by flow cytometry after exposure to AG-QDs (50, 100, and 200 μg/mL). The areas denoted as R1, R2, R3, and R4 in Fig. 2a-2b indicate viability, early apoptosis, late apoptosis, and necrotic cells, respectively. The proportions of apoptotic cells after 24-h exposure to AG-QDs at 100 and 200 μg/mL were 8.30% and 22.0%, respectively; these values are significantly higher than that of unexposed cells (2.13%) (*p* < 0.05). After 48-h exposure, significant increases in apoptotic cells were observed for AG-QDs at 100 and 200 μg/mL. In addition, early apoptosis was evident at all exposure times and AG-QDs concentrations (Fig. 2c). Importantly, early apoptosis was AG-QDs concentration-and time-dependent; significant increases in early apoptosis were observed after exposure to AG-QDs (100 μg/mL) for 24 and 48 h (*p* < 0.05) (Additional file 1: Figure S5). Less than 3% of cells were observed to be in necrosis across all the treatments (Fig. 2d), indicating that AG-QDs induced cell death was primarily due to apoptosis.

### Uptake and exocytosis processes of AG-QDs

**Fig. 3:**
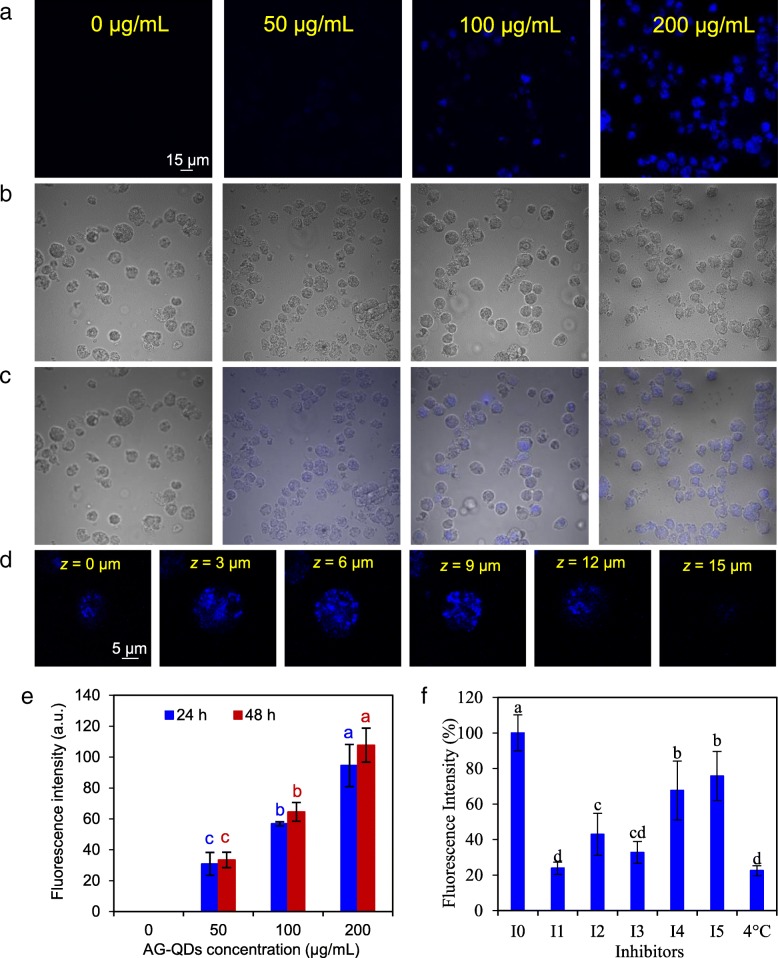
Uptake of AG-QDs by NR8383 cells under confocal imaging and analysis. **a**, **b**: Confocal images of NR8383 cells after treatment with AG-QDs (0, 50, 100, and 200 μg/mL) for 24 h under fluorescence excitation, and bright field, respectively. **c**: Merged images of (**a**) and (**b**). **d** Fluorescence intensity of AG-QDs (200 μg/mL) in NR8383 cell at different cell depths along the *z* axis (*z* = 0, 3, 6, 9, 12, and 15 μm). **e** Uptake (indicated by blue fluorescence) of AG-QDs (0, 50, 100 and 200 μg/mL) after exposure for 24 and 48 h as quantified by a fluorescence spectrophotometer. **f** Effect of specific endocytosis inhibitors on the uptake of AG-QDs (200 μg/mL) by NR8383 cells during 24-h incubation. In panel (**f**), the treatments I0-I5 represent the cells after incubation with AG-QDs, NaN_3_, cytochalasin D, genistein, chlorpromazine, and amiloride, respectively. Significant differences among different treatments are marked with letters “a-d” (*p* < 0.05, LSD, *n* = 6). In panel (**e**), for a given exposure time, significant differences among AG-QDs concentrations are marked with different letters (*p* < 0.05, LSD, *n* = 6)

The cellular uptake of AG-QDs was detected by confocal laser scanning microscopy (CLSM). Un-exposed NR8383 cells showed no fluorescence signals; the intracellular blue fluorescence intensity increased with increasing AG-QDs exposure concentrations (50, 100 and 200 μg/mL), indicating that cellular uptake was concentration-dependent (Fig. 3a-3c). A series of confocal images along the *z* axis of NR8383 cells were further imaged to exclude possible attachment of AG-QDs on the cell surface (Fig. 3d). The fluorescence intensity gradually increased and achieved a maximum at the medium depth (~ 9 μm) of cells, confirming cellular internalization of AG-QDs. The quantitative evaluation of AG-QDs internalization in NR8383 cells is shown in Fig. 3e. After exposure to AG-QDs at 200 μg/mL for 24 h, the intracellular AG-QDs content was 3.07 and 1.67 times higher than that at 50 and 100 μg/mL, respectively. At a given AG-QDs concentration, there was no significant difference between 24- and 48-h exposure, suggesting that uptake had occurred in less than 24 h.

As a “first responder”, macrophages primarily use endocytosis when encountering foreign materials [13]. The main endocytotic pathways were investigated in the current study by using five inhibitors, including NaN_3_, cytochalasin D, chlorpromazine, amiloride, and genistein (Fig. 3f). The fluorescence intensity of AG-QDs in NR8383 cells was significantly decreased for all tested endocytosis inhibitors (*p* < 0.05). NaN_3_ (energy-dependent endocytosis inhibitor), cytochalasin D (phagocytosis inhibitor) and genistein (caveolae-mediated endocytosis inhibitor) showed much greater reduction in AG-QDs uptake than chlorpromazine (clathrin-mediated endocytosis inhibitor) or amiloride (macropinocytosis inhibitor). These results demonstrate that energy-dependent endocytosis, phagocytosis and caveolae-mediated endocytosis play a more important roles in the intracellular accumulation of AG-QDs by NR8383 cells. In addition, the internalization of AG-QDs was highly inhibited at low temperature (4 °C) (Fig. 3f), demonstrating that cellular uptake of AG-QDs was energy-dependent.

Cellular export is an important process for AG-QDs fate after internalization by macrophages. We thus quantified particle excretion after incubation with AG-QDs (50, 100 and 200 μg/mL) for 24 h (Additional file 1: Figure S6a). For each pretreatment concentration, the released amount of AG-QD increased with increasing excretion times. After excretion for 48 h, intracellular AG-QDs was reduced by 23.3–35.2%, confirming particle export by the macrophages. This observed excretion phenomenon could be a cellular detoxification pathway post-AG-QDs exposure [23]. However, a significant fraction of AG-QDs remained in cellular cytoplasm and nucleus even after 48-h excretion (Additional file 1: Figure S6b), which was confirmed by using SYTO 9 probes to identify AG-QDs in the nuclei (Additional file 1: Figure S7). It has been reported that the distribution and entrapment of nanoparticles (NPs) into cytoplasm and nucleus are the limiting processes for exocytosis [23, 24]. The translocation and fate of AG-QDs in macrophage nuclei was thus further investigated.

### Accumulation of AG-QDs in cell nucleus

**Fig. 4:**
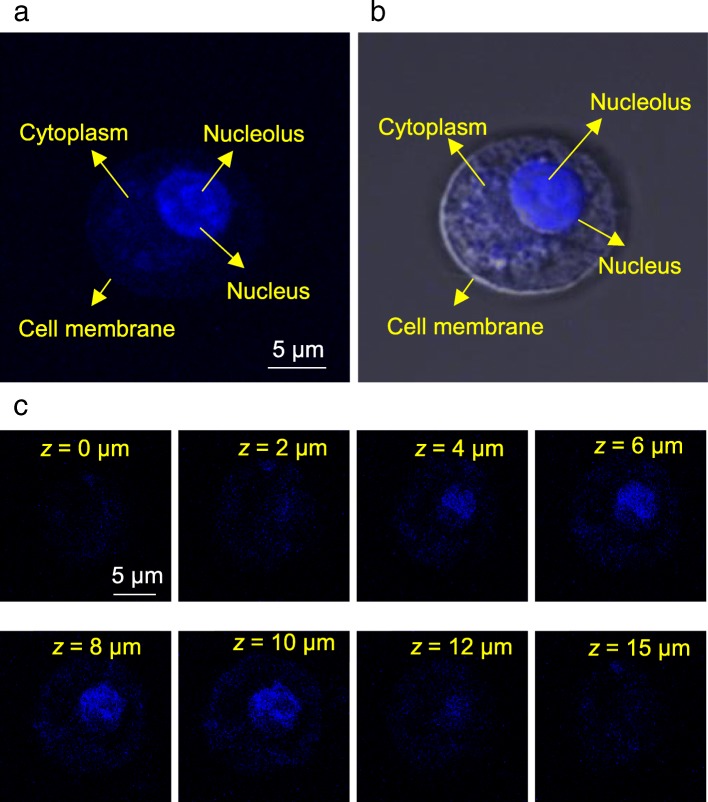
Accumulation of AG-QDs in the nucleus as imaged by confocal microscopy The NR8383 cells were incubated with AG-QDs (200 μg/mL) for 24 h before imaging. **a**: Fluorescence image under 405-nm excitation and 438-nm emission (blue). **b**: The merged fluorescence and bright field images. **c**: Fluorescence intensity of AG-QDs in NR8383 cells (as shown in panel (**a**)) at different cell depths along the *z* axis (*z* = 0, 2, 4, 6, 8, 10, 12 and 15 μm)

After cellular internalization, AG-QDs could distribute in mitochondria, endo-lysosomes and endoplasmic reticulum of NR8383 cells (Additional file 1: Figure S8). These AG-QDs in endo-lysosomes could escape into the cytoplasm of NR8383 cells as indicated by a stability decrease of endo-lysosomes membrane (Additional file 1: Figure S9). Interestingly, for most of macrophages evaluated after 24-h AG-QDs exposure, the fluorescence intensity of cell nuclei was much stronger than other cellular areas (Fig. 4a, b), suggesting significant internalization of the particles in this important organelle. *z*-Axis imaging of NR8383 cells after 24-h exposure was conducted (Fig. 4c). AG-QDs are clearly present in NR8383 cells along the *z* axis (*z* = 0–15 μm), with the maximum fluorescence intensity occurring at a depth of 8–10 μm in the cells. To examine the role of -NH_2_ on AG-QDs, another type of graphene QDs (GO-QDs) without -NH_2_ groups (characterization data in Additional file 1: Figure S10) was employed for the cellular/nuclear distribution assay. It is shown that GO-QDs were internalized in NR8383 cells, and were accumulated in nuclei after 24-h exposure (Additional file 1: Figure S10), suggesting that the observed nuclear localization was not dependent on the -NH_2_ groups on AG-QDs. Interestingly, the nuclear uptake process was exposure time-dependent. After exposure for 12 h, AG-QDs were mainly located in the cytoplasm while insignificant fluorescence signals were detected in the nucleus (Additional file 1: Figures S11 and S12). To further investigate the time-dependent nuclear uptake of AG-QDs, the expression of two key nuclear pore complex (NPC) genes, karyopherin *β*2 (*Kapβ2*) and nucleoporin 98 (*Nup98*), was determined. *Kapβ2* is a prototypical *Kapβ*, which binds important substrates and nucleoporins simultaneously for nuclear transfer [25, 26]. *Nup98* plays a critical role in regulating the permeability barrier that inhibits macromolecular diffusion [27]. The expression *Kapβ2* was down-regulated after AG-QDs exposure for 12 h in comparison to the un-exposed group (Additional file 1: Figure S13). The down-regulation of *Kapβ2* suggests an inhibition of the biochemical selectivity of the nuclear envelope. On the contrary, the expression of *Nup98* was significantly increased after 12-h exposure (*p* < 0.05), demonstrating that the passive permeability barrier was activated, serving to inhibit AG-QDs diffusion from cytoplasm to nucleus. Interestingly, the regulation of both genes returned to normal after 24-h exposure and was not significantly different from the un-exposed group (Additional file 1: Figure S13); this is consistent with our previous finding that nuclear distribution of AG-QDs was observed only after 24-h exposure. Therefore, it is very likely that the nuclear uptake of AG-QDs was regulated by *Kapβ2* and *Nup98* activities. However, it is still unknown if *Kapβ2* and *Nup98* were also triggered by AG-QDs before nuclear uptake.

### Nuclear damage by AG-QDs

**Fig. 5:**
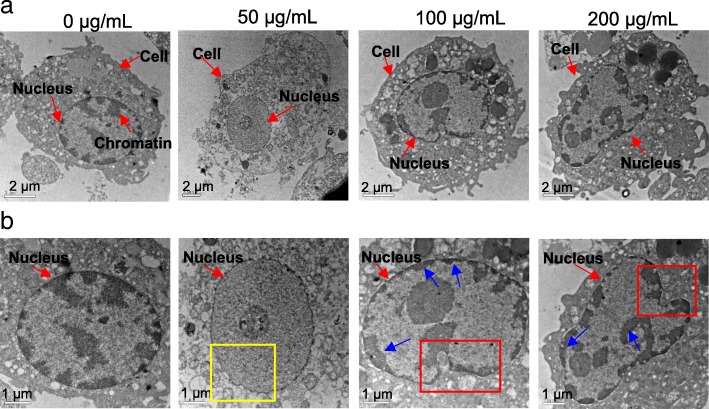
TEM images of NR8383 nuclear morphology after exposure to AG-QDs (0, 50, 100, and 200 μg/mL) for 24 h. The images in Panel (**b**) are enlarged from panel (**a**). In panel (**b**), the yellow box indicates the shrinking of the inner nuclear envelope after AG-QDs (50 μg/mL) exposure. The red boxes indicate the malformation of nuclear morphology after AG-QDs (100 and 200 μg/mL) exposure. The blue arrows indicate the chromatin condensation (electron-dense, black structure along nuclear membrane) within the nuclei

**Fig. 6:**
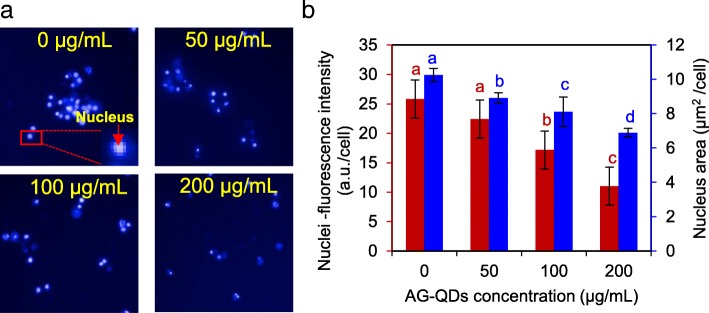
Effect of AG-QDs on nuclear viability and area as detected by high content screening. **a**: High content screening images of nucleus (as stained by Hoechst 33342). The NR8383 cells were exposed to AG-QDs (0, 50, 100 and 200 μg/mL) for 24 h before staining and imaging. The stained cell at the bottom right corner is magnified from the cell marked with red box. **b** Viability (red) and area (blue) of nuclei as obtained from quantitative analysis of the intensity and area of nuclei fluorescence in panel (**a**). Significant differences among different AG-QDs concentrations (0–200 μg/mL) is marked with different letters (*p* < 0.05, LSD, *n* = 6)

Morphological changes of cell nucleus after AG-QDs exposure were characterized using TEM (Fig. 5). The nucleus of unexposed cells was intact and elliptical in shape, with randomly distributed chromatins. After exposure to AG-QDs (50 μg/mL) for 24 h, shrinking of the inner nuclear envelope was observed (as indicated by the yellow box in Fig. 5b). At higher AG-QDs concentrations (100 and 200 μg/mL), nuclear morphology became more irregular and malformed. In addition, the chromatins in nucleus were highly condensed, and were mainly attached on nuclear membrane or in the form of long-chain structures (blue arrows in Fig. 5b). We employed the High Content Screening (HCS) to further investigate the changes in nuclear morphology and related viability. The HCS images on the Hoechst-stained nuclei are showed in Fig. 6a, and the influence of AG-QDs on the fluorescence intensity of Hoechst-stained nuclei was negligible (Additional file 1: Figure S14). The quantitative results show that nuclear areas were significantly decreased with increasing AG-QDs concentrations (Fig. 6b), confirming the observed shrinkage of nuclei in Fig. 5b. Moreover, fluorescence intensities of Hoechst-stained nuclei were significantly decreased after exposure to AG-QDs at 100 and 200 μg/mL (Fig. 6a, b), indicating a reduction in nuclear viability.

Nuclear areas of NR8383 cells were evaluated using Raman spectroscopy to identify organelle components impacted by AG-QDs. The microscopic images marked with red squares were analyzed (Additional file 1: Figure S15a-S15c), and the corresponding Raman spectra are shown in Additional file 1: Figure S15d. A Raman peak at 790 cm^− 1^ represents the vibration of tyrosine and cytosine on the DNA backbone, while 1003, 1098, 1768, and 2429 cm^− 1^ indicate the vibration of phenylalanine, DNA PO_2_^−^ backbone, lipids C=O, and protein C=N, respectively [28]. The vibration strengths on DNA tyrosine and cytosine (790 cm^− 1^), and PO_2_^−^ backbone (1098 cm^− 1^) decreased with increasing exposure times (0, 24, and 48 h), clearly suggesting the disruption on DNA structure [29]. Moreover, the peaks (e.g., D and G bands) of AG-QDs were too weak to observe in NR8383 cells after AG-QDs exposure.

### Mechanisms on DNA cleavage induced by AG-QDs

**Fig. 7:**
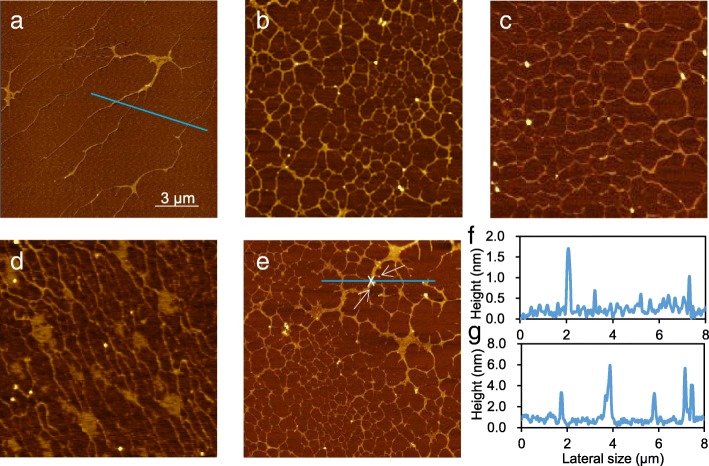
Investigations on DNA chain damage caused by AG-QDs. **a**, **b**: AFM images of DNA morphology in unexposed NR8383 cells and the cells after exposure to AG-QDs (200 μg/mL) for 24 h. The DNA chains were extracted from cells before imaging. **c**, **d**: AFM images of DNA morphology in NR8383 cells after exposure to H_2_O_2_ (50 μL/mL), AG-QDs (200 μg/mL) with GSH (1 mg/mL) for 24 h. **e**: AFM images of DNA chains that were directly exposed to AG-QDs (200 μg/mL) for 24 h. **f**, **g**: The height profiles of DNA chains as marked on the AFM images in panels (**a**) and (**e**), respectively. In panel (**e**), white arrows indicate the particles on DNA chains

The disruption of DNA chains caused by AG-QDs was characterized using atomic force microscopy (AFM). A characteristic long-chain structure was clearly observed for the extracted DNA samples from the normal unexposed NR8383 cells (Fig. 7a). Interestingly, for the DNA extracted from the AG-QDs exposed cells, the typical long-chain structures were cleaved into the shorter-chain and cross-linked structures (Fig. 7b). Oxidative damage, direct physical contact, and the up-regulation of caspase genes are three possible reasons for this observed disruption of DNA chains. We thus first investigated the over-generation of ROS in NR8383 cells during AG-QDs exposure. It is shown that AG-QDs alone did not produce ROS, while intracellular ROS level significantly increased after exposure to AG-QDs (200 μg/mL) for 24 h (Additional file 1: Figure S16). These results confirmed the oxidative stress of AG-QDs on cells. Then, H_2_O_2_ (oxidizing agent) and GSH (antioxidant) were used to verify oxidative DNA damage induced by AG-QDs (200 μg/mL) exposure. After exposure to H_2_O_2_ for 24 h, the extracted DNA showed similar cleavage and cross-linking (Fig. 7c). In the presence of GSH, the degree of DNA cleavage and cross-linking from AG-QDs-exposed cells was notably mitigated (Fig. 7d), confirming that oxidative stress had occurred, and that oxidative DNA damage was an important mechanism for DNA disruption after particle exposure.

After nuclear uptake, the accumulated AG-QDs may bind with DNA chains, causing physical damage by direct contact. An investigation evaluating the direct interaction between AG-QDs and pre-extracted DNA chains was conducted. Clearly, significant cleavage and cross-linking occurred for the AG-QDs-contacted DNA chains in DI water (Fig. 7e). The particles (indicated with white arrows) were observed on the DNA breaking points (Fig. 7e); the height of these particles was ~ 6 nm (Fig. 7g). The normal thickness of DNA chains was only 1–2 nm (Fig. 7f) while the lateral size of the AG-QDs was ~ 4.1 nm (Fig. 1a), indicating the presence of AG-QDs that had adsorbed or intercalated on DNA chains. This finding is consistent with that of Ren et al., in which the nano-sized GO sheets were reported to bind to the DNA in an intercalative manner [30]. Because AG-QDs may be still coated with FBS during direct contact with DNA chains in NR8383 cells, the direct interaction between AG-QDs-FBS and DNA chains were investigated (Additional file 1: Figure S17). The height of the particles (indicated with white arrows) observed on the DNA chains were 6.6 and 10.4 nm; the height of AG-QDs in FBS was 4.30–10.2 nm (Fig. 1d), indicating that AG-QDs coated with protein corona could also adsorb or intercalate on DNA chains. Moreover, AG-QDs induced similar cleavage and cross-linking of DNA chains in the presence of FBS, confirming that the DNA cleavage could be caused by the internalized AG-QDs in NR8383 cells, and this effect is independent of the coating of FBS.

**Fig. 8:**
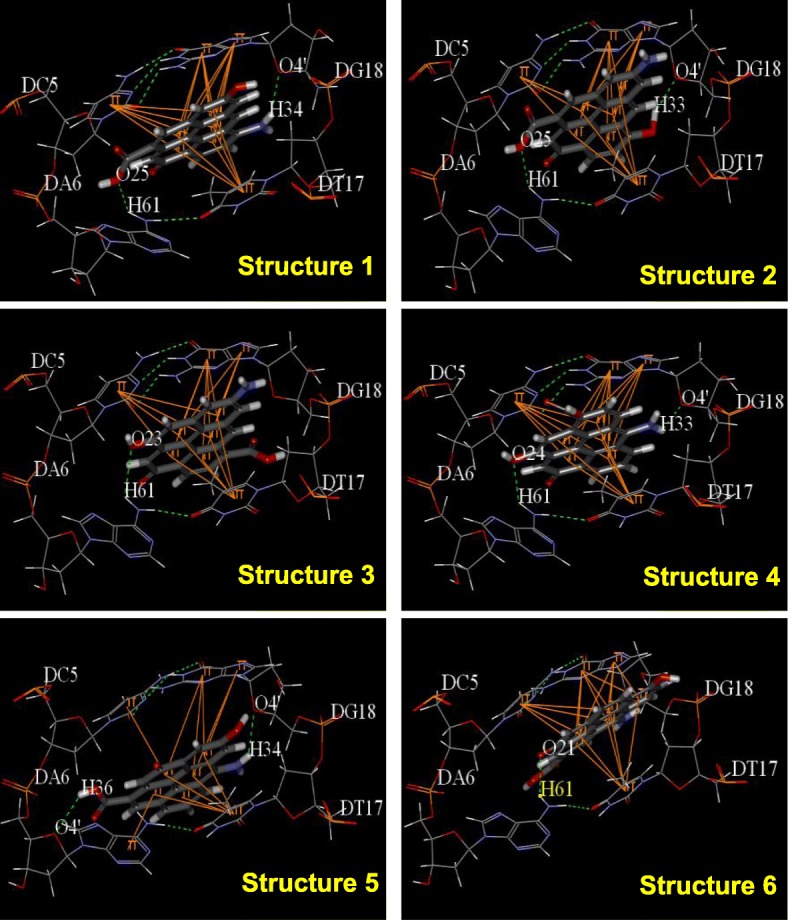
Hydrogen bonding and π-π interactions between AG-QDs and DNA at select binding sites. Green dotted lines show H-bonds between AG-QDs and basic groups, purines and pyrimidines. Yellow lines show π-π interactions between AG-QDs and basic groups (cytimidine, guanine, adenine, and thymine). Carbon, oxygen, hydrogen, and nitrogen are colored in grey, red, white, and blue, respectively

Molecular docking was then employed to explore the interaction mechanisms between AG-QDs and DNA chains. During the docking analysis, 10 structural models (Additional file 1: Figure S18) of AG-QDs containing representative functional groups (e.g., -NH_2_, -COOH, -OH, O=C-) based on XPS data (Additional file 1: Figure S1b) were used to simulate the interaction forces between AG-QDs and DNA. Among these 10 AG-QDs structures, 6 were able to form different kinds of H-bonds with DNA bases/deoxyribose: (a) H-bonds formed between the amino hydrogen (H^33, 34^) of AG-QDs (Structures 1, 4 and 5) and the oxygen (O^4^) of deoxyribose; (b) H-bonds between the carboxyl oxygen (O^25^) of AG-QDs (Structures 1, and 2) and the amino hydrogen (H^61^) of adenine; (c) H-bonds between the hydroxyl hydrogen (H^33^) of AG-QDs (Structure 2) and the oxygen (O^4^) of deoxyribose; (d) H-bonds between the hydroxyl oxygen (O^23, 24^) of AG-QDs (Structures 3, and 4) and the amino hydrogen (H^61^) of adenine; (e) H-bonds between the carboxyl hydrogen (H^36^) of AG-QDs (Structure 5) and the oxygen (O^4^) of adenine; (f) H-bonds between the double-bonded oxygen (O^21^) of AG-QDs (Structure 6) and the amino hydrogen (H^61^) of adenine (Fig. 8). In addition, π-π stacking was observed between the benzene rings of DNA bases (e.g., A, T, G, and C) and all 10 AG-QDs structural models (Fig. 8, Additional file 1: Figure S19). In addition, the number of π bonds between AG-QDs (Structures 1–10) and DNA chains was quantified (Additional file 1: Table S1). For each AG-QDs structure, 11 or more π bonds were formed with DNA bases. It is well known that the DNA double helix is stabilized primarily by two forces: (1) hydrogen bonding between nucleotides, and (2) base-stacking interaction among aromatic nucleobases. The hydrogen bonding and π-π stacking between model AG-QDs and DNA chains (Fig. 8) may lead to the disruption of the DNA double helix, subsequently causing the observed cleavage and cross-linking (Fig. 7).

The activation of caspases can lead to cell apoptosis and may also be involved in DNA cleavage [31]. Therefore, RNA sequencing (RNA-Seq) was used to investigate the expression of cellular caspases upon AG-QDs exposure. The caspase gene family in *Rattus norvegicus* genome contains 10 members (*caspase-1*, *− 2*, *− 3*, *− 4*, *− 6*, *− 7*, *− 8*, *− 9*, *− 12*, and *− 14*), in which the genes of *caspase-1*, *− 4*, *− 7*, and *− 8* were up-regulated after AG-QDs exposure for 24 h; activation of other 6 caspase genes was not observed (Additional file 1: Table S2). Caspase-activated DNase (CAD) is a protein that can break the DNA chain during apoptosis. Under apoptotic conditions (previously observed in Fig. 2), the activation of caspases could hydrolyze the inhibitor of CAD (ICAD), dissociate the CAD/ICAD heterodimer, release CAD from cytosol to the nucleus, and cause cleavage of DNA chains [32]. The activation of *caspase-1* was reported to stimulate nuclease activity and induce the DNA cleavage in macrophages (J774A.1) after the infection by *Salmonella enterica* serovar Typhimurium [33]. The up-regulation of *caspase-4*, *− 7* and *− 8* could also induce the CAD/ICAD inactivation and DNA cleavage [34, 35]. Therefore, the up-regulation of *caspase-1*, *− 4*, *− 7*, and *− 8*, and subsequent apoptosis also played important role in the observed DNA damage.

## Discussion

In the present work, AG-QDs (lateral size, 4.1 nm) had a higher 24-h MIC value (200 mg/L) to macrophages (Additional file 1: Figure S3) than does graphene (lateral size, ~ 200 nm) (MIC, 50 μg/mL) [36], GO (lateral size, 300–750 nm) (MIC, 20 μg/mL) [37], or amino-functionalized carbon quantum dots (lateral size, 10 nm) (MIC, 100 μg/mL) [38], suggesting that smaller lateral size could be a main reason for the lower cytotoxicity of AG-QDs [39]. This finding is different from the toxicity to algal cells (*Chlorella vulgaris*), in which GO-QDs (lateral size, 20–50 nm) induced higher toxicity because of higher internalization than the normal GO sheets (lateral size, 1–5 μm) [40]. This low cytotoxicity highlights the possibility for safe application of AG-QDs in biomedicine. In addition, AG-QDs induced a significant increase of apoptotic cell and negligible change of necrotic cell (Fig. 2, Additional file 1: Figure S5). It is observed that early apoptosis was evident at all exposure times and AG-QDs concentrations. It is well known that early stage apoptosis can promote the loss of membrane integrity, compromise the mitochondrial membrane, and even stimulate programmed cell death [41]. Taken together, it is clear that early apoptosis plays a major role in AG-QDs-induced cell death, and the toxicity of AG-QDs cannot be neglected.

Our results demonstrated that AG-QDs could be internalized by macrophages, and cellular uptake of AG-QDs was primarily by energy-dependent endocytosis, phagocytosis and caveolae-mediated endocytosis (Fig. 3f). NaN_3_ and low temperature (4 °C) significantly inhibited AG-QDs uptake because endocytotic processes including phagocytosis, pinocytosis, caveolae-mediated, and clathrin-mediated endocytosis are all energy-dependent [42]. Phagocytosis is a specialized process by which macrophages engulf large or aggregated particles (< 10 μm); given that AG-QDs formed larger aggregates during the uptake assay, phagocytosis is likely an important pathway for AG-QDs internalization. CdTe QDs were reported to be taken up by macrophages (Kupffer cells) via phagocytosis [43], consistent with our findings. In the present work, AG-QDs were detected within endo-lysosomes (Additional file 1: Figure S8), confirming the phagocytosis pathway. It is reported that peptides (e.g., histidine-rich peptides) coated on the surface of NPs could destabilize endo-lysosomes membrane, which is an important mechanism for the escape of NPs from endo-lysosomes [44]. Actually, the integrity of endo-lysosomes membrane was decreased by AG-QDs (Additional file 1: Figure S9). Therefore, in our case, AG-QDs are likely to escape from endo-lysosomes with the assistance of adsorbed FBS, and then be taken up by nuclei. But this escape pathway needs further investigation. Caveolae-mediated endocytosis could bypass the endosomes and lysosome vesicles, transporting the NPs (< 80 nm) directly to endoplasmic reticulum and nucleus [45]. Wu et al. reported that caveolae-mediated endocytosis was a significant pathway for graphene QDs (~ 20 nm in lateral size) internalization in MCF-7 cells [7], which is in good agreement with our result that AG-QDs were efficiently transported via caveolae-mediated endocytosis.

A major finding presented here is that AG-QDs after cellular uptake were highly accumulated in nucleus, which is in good agreement with another result that less than half of the internalized AG-QDs were exported from NR8383 cells after a 48-h excretion period. It has been reported that nano-sized particles (∼10 nm) can passively diffuse into nuclei through NPCs (10 nm in pore diameter) [46]. In the present work, a large proportion of AG-QDs were smaller than 10 nm even after being coated with serum proteins (Fig. 1), suggesting that passive diffusion is the primary nuclear uptake pathway for these particles. Furthermore, two NPC genes including *Kapβ2 Nup98* were shown to regulate nuclear uptake of AG-QDs during the passive diffusion of AG-QDs. In addition, it is confirmed that nuclear uptake of AG-QDs (lateral size, 4.1 nm; O/C atomic ratio, 0.525) was independent of their -NH_2_ groups on the surface (Additional file 1: Figure S10). Another study reported that graphene QDs (lateral size, 3.2~ 3.6 nm) with different functional groups (O/C atomic ratio, 0.150–0.275) did not diffuse into the nucleus of osteoblastic cells (MC3T3-E1) [10]. Higher O/C atomic ratio associated with lower aggregation is likely a reason for the nuclear uptake of AG-QDs in this work. The observed nuclear uptake may be also cell type-dependent (macrophages *v.s.* normal mammalian cells), which needs to be further verified.

TEM imaging showed that AG-QDs in nucleus induced the shrinkage and malformation of nuclear morphology and unevenly-distribution of chromatins (Fig. 5). An irregular shape and the unevenly-distributed chromatins in glioblastoma cells were also observed after GO exposure, in agreement with our findings of NPs-induced alteration of nuclear morphology [47]. Nuclear malformation and chromatin condensation are known hallmarks of apoptosis [48], which have occurred in the exposed NR8383 cells as shown in Fig. 2. Accumulated AG-QDs in nuclei also resulted in the decrease of nuclear areas and the reduction of nuclear viability upon HCS analysis (Fig. 6). Disruption of the DNA backbone was further detected in the AG-QDs treated cells by Raman spectra (Additional file 1: Figure S15). These results suggested that AG-QDs could potentially cause nuclear damage after environmental exposure although it is low-toxic via growth inhibition assay.

Importantly, it is demonstrated that AG-QDs could cause significant DNA chain cleavage and cross-linking in NR8383 cells. Three mechanisms including oxidative damage, direct contact, and the up-regulation of caspases were responsible for the observed disruption of DNA chains by AG-QDs. Oxidative DNA damage was confirmed after AG-QDs exposure in the present work. Intracellular reactive oxygen species (ROS) (e.g., •OH) could be a major contributor to DNA phosphate backbone cleavage through hydrogen abstraction from the deoxyribose sugar [49]. Kim and Kang also observed that •OH generated in the Fenton-like reaction of cytochrome c/H_2_O_2_ system induced the cleavage of plasmid DNA [50]. Therefore, •OH generation could also be an important cause for the observed DNA damage upon NPs exposure in the present work. In addition, the accumulated AG-QDs in nucleus could directly contact with DNA chains and cause physical DNA damage as observed from atomic force microscopy imaging. H-bonding and π-π stacking played dominant forces during the interactions between AG-QDs and DNA chains via molecular docking simulation (Fig. 8, Additional file 1: Figure S19), which disrupted the stabilized DNA double helix, subsequently causing the observed cleavage and cross-linking. Our previous study also showed the disruption of DNA nanostructures by the benzene ring- and hydroxyl-containing Bisphenol A through hydrogen bonding and π-π stacking [51]. Furthermore, H-bonding between AG-QDs and DNA bases may either block DNA replication and transcription or generate mutations by miscoding during replication [52]. π-π Stacking could inhibit gene expression and cellular differentiation, and promote cellular apoptosis through disrupting double-helix structure of DNA [53]. Electrostatic attraction has been reported to contribute to the adsorption between DNA chains and NPs such as Au, ZnO, and Fe_3_O_4_ [54–56]. However, both AG-QDs and DNA chains are negatively charged; thus, electrostatic attraction could not be a dominant force for AG-QDs-DNA interaction in our system. The activation of caspases leading to cell apoptosis could also induce DNA cleavage. The genes of *caspase-1*, *− 4*, *− 7*, and *− 8* in NR8383 cells were up-regulated after AG-QDs exposure for 24 h by RNA sequencing (Additional file 1: Table S2). The activation of *caspase-1*, *− 4*, *− 7*, and *− 8* were reported to stimulate nuclease activity and induce the DNA cleavage [32–34], which were in good agreement with our present results. All these results suggested that graphene QDs could be accumulated in nuclei of macrophages, and the investigation on nuclear DNA damage brings new insight into genotoxicity of graphene QDs.

## Conclusions

It is observed that AG-QDs exhibited low overt cytotoxicity to NR8383 cells (MIC, 200 μg/mL), but induced a significant increase of cell apoptosis, with early apoptosis playing a major role in the AG-QDs-induced cell death. AG-QDs were internalized primarily by energy-dependent endocytosis, phagocytosis and caveolae-mediated endocytosis. Significant amounts of the particles would be retained in the cytoplasm and nucleus after a 48-h excretion period. The internalized AG-QDs were accumulated in nucleus; the NPC genes *Kapβ2* and *Nup98* were shown to regulate nuclear uptake of AG-QDs. AG-QDs in nucleus altered nuclear morphology, decreased nuclear areas, and reduced nuclear viability. Disruption of the DNA backbone was also detected after AG-QDs exposure. It is demonstrated that AG-QDs could cause significant DNA chain cleavage and cross-linking. Oxidative damage, direct contact via H-bonding and π-π stacking, and the up-regulation of caspases are the primary mechanisms for the observed disruption of DNA chains by AG-QDs. These findings advance our understanding of the potential nuclear toxicity and DNA damage mediated by AG-QDs uptake and accumulation in macrophages, and will provide useful knowledge for health risk assessment of this unique nanoparticle.

## Methods

### AG-QDs characterization

AG-QDs were purchased from Nanjing XFNANO Materials Tech Co., Ltd. (China). Elemental analysis of AG-QDs was conducted by X-ray photoelectron spectroscopy (XPS) (ESCALAB 250Xi, Thermo scientific, USA). Particle morphology in both DI water and culture medium (F12 K medium supplemented with 15% FBS) was examined by transmission electron microscopy (TEM) (H-7650, Hitachi, Japan) and atomic force microscopy (AFM) (Agilent-5400, USA). The zeta potential and hydrodynamic diameter of AG-QDs in DI water (50 μg/mL) and cell culture medium (0, 50, 100, and 200 μg/mL) were determined on a Zetasizer (ZS90, Malvern, Britain). In addition, fluorescence spectra of the particles were recorded by a fluorescence spectrophotometer (Hitach F-4500, Japan) with an emission wavelength at 438 nm.

### Cell culture and viability assay

The NR8383 cell line was purchased from Shanghai Institute for Biological Sciences, Chinese Academy of Science. NR8383 cells were cultured at 37 °C in F12 K medium (Sigma-Aldrich, St. Louis, MO) supplemented with 15% FBS (PAA Laboratories GmbH, Austria), 2 mM L-glutamine (Amresco Inc., USA), and 1% penicillin/ streptomycin (Haoyang Biological Manufacture Co., Tianjin, China) in an incubator with 5% CO_2_ [23]. MC3T3-E1 cells (iCell Bioscience Inc., Shanghai, China) were cultured at 37 °C in Dulbecco’s modified Eagle’s medium (DMEM) (Sigma-Aldrich, St. Louis, MO) supplemented with 10% FBS in an incubator with 5% CO_2_.

Cell viability was determined by Cell Counting Kit-8 (CCK-8, Beyotime Institute of Biotechnology, China). Briefly, NR8383 (1 × 10^7^ cells/mL) or MC3T3-E1 cells (1 × 10^5^ cells/mL) in 96-well plates were exposed to different concentrations of AG-QDs (0, 10, 25, 50, 100, 200, and 500 μg/mL) at 37 °C for 12, 24, 48, 72 and 96 h, and were then treated with CCK-8 probes to determine cell viability with a microplate reader (Thermo-1500, USA).

### Cell apoptosis and necrosis assay

Upon exposure, apoptotic and necrotic cells were detected with the FITC Annexin V apoptosis kit (Beyotime Institute of Biotechnology, China). NR8383 cells (1 × 10^7^/mL) were first exposed to AG-QDs at different concentrations (0, 50, 100 and 200 μg/mL) for 24 and 48 h. The cells were then washed three times with phosphate buffered saline (PBS). The washed cells were re-suspended in 200 μL Annexin V-FITC binding buffer, and were stained with Annexin V-FITC (5 μL) and propidium iodide (PI, 5 μL). After incubation for 20 min at 25 °C in the dark, the apoptotic and necrotic cells were assessed by flow cytometry (Becton Dickinson, Mountain View, USA). The exposed cells at both early and late apoptosis were indicated by FITC labeled Annexin V. PI indicated damage of cell membrane, which occurs in late apoptosis and necrosis. The early apoptosis and late apoptosis cells were identified as Annexin V+/PI− and Annexin V+/PI+, respectively. The necrotic cells were identified as Annexin V–/PI+ and viable cells were identified as Annexin V–/PI−.

### Cellular uptake and exocytosis of AG-QDs

After exposure to AG-QDs (0, 50, 100 and 200 μg/mL) for 12 and 24 h, the NR8383 cells were washed three times with PBS buffer. The uptake of AG-QDs in NR8383 cells was then investigated by confocal laser scanning microscopy (CLSM) (FV1000, Olympus, Japan) at 405 nm excitation and 438 nm emission. A series of confocal images of the whole cells and cell nuclei were captured at different depths (*z* axis). The fluorescence intensity of AG-QDs in cells was quantified by fluorescence spectrophotometer (Hitach F-4500, Japan). For comparison, cellular uptake of GO-QDs (Nanjing XFNANO Materials Tech Co., Ltd., China) was also investigated by following the above approaches. The distribution of AG-QDs in mitochondria, endo-lysosomes, and endoplasmic reticulum was further examined. Briefly, NR8383 cells after exposure to AG-QDs (200 μg/mL, 24 h) were washed with PBS. Nuclei and mitochondria/endo-lysosomes/endoplasmic reticulum in NR8383 cells were co-stained with SYTO 9 (1 μM) and Mito-Tracker Red (100 nM)/Lyso-Tracker Red (50 nM)/ER-Tracker Red (300 nM) to indicate nuclei and mitochondria/endo-lysosomes/endoplasmic reticulum, respectively. The locations of nuclei (green fluorescence, 500 nm excitation and 530 nm emission) and mitochondria (red fluorescence, 587 nm excitation and 615 nm emission), endo-lysosomes (red fluorescence, 587 nm excitation and 615 nm emission), or endoplasmic reticulum (red fluorescence, 587 nm excitation and 615 nm emission) were then observed and imaged by CLSM.

To analyze the endocytotic pathway, specific inhibitors including NaN_3_ (3 mM), cytochalasin D (10 μM), genistein (200 μM), chlorpromazine (10 μg/mL), and amiloride (50 μg/mL) were employed during AG-QDs (200 μg/mL) exposure at 37 °C. In addition, low-temperature (4 °C) incubation was carried out for the AG-QDs (200 μg/mL) exposure assay. For the exocytosis assay, NR8383 cells pre-treated with AG-QDs (200 μg/mL, 24 h) were washed three times with PBS, and then re-cultured in fresh media. After re-culturing for 2, 6, 12, 24, or 48 h, the cells were observed with CLSM. The fluorescence intensities were determined with a fluorescence spectrophotometer.

### Quantitative real-time PCR (qRT-PCR) analysis

After exposure to AG-QDs (200 μg/mL) for 12 and 24 h, NR8383 RNA was extracted using Trizol according to the manufacturer’s instructions (Tiangan Biotech CO., China). The mRNA levels for the genes of interest (*Kapβ2* and *Nup98*) were determined using SYBR Green (FP205, TIANGEN Biotech CO., LTD., China) by qRT-PCR (Mx3005P, Bio-Rad, USA). The detailed procedures were previously reported [23]; the primers for the PCR reactions are listed in Additional file 1: Table S3.

### Nuclear morphology, area and viability

Morphological changes of the AG-QDs exposed-nuclei were characterized by TEM. NR8383 cells that had been exposed to AG-QDs (0, 50, 100 and 200 μg/mL) for 24 h were collected and washed with PBS buffer. The fixation, dehydration and embedding of samples were conducted by following the procedures of Wang et al. [23]. The ultrathin sections were obtained by Ultra microtome (UC7, Leica, Germany) before TEM imaging.

High content screening (HCS) was used to examine the nuclear area and viability of NR8383 cells after AG-QDs exposure. Briefly, NR8383 cells were exposed to AG-QDs (0, 50, 100, and 200 μg/mL) for 24 h. The cells were fixed with 4% (*w*/*v*) paraformaldehyde (100 μL, 30 min) and were permeated with 0.1% Triton X-100 (*v*/v) in PBS buffer (100 μL, 30 min). The nuclei were stained by 100 μL Hoechst 33342 (3 μg/mL) for 1 h, and the collected cells were further stained with 100 μL Alexa 488 (0.2%, v/v) for 1 h. The cells were washed with PBS buffer for each staining procedure. The nuclear viability was determined as indicated by the fluorescence intensity of Hoechst-stained nuclei, and the nuclear area of the stained cells were obtained by HCS (PerkinElmer, USA).

### Investigations on interaction between DNA and AG-QDs

A laser Raman microscope (Thermo Fisher, USA) was used to investigate the alteration of nuclear components after uptake of AG-QDs (200 μg/mL) into this organelle for 24 and 48 h. The nuclear areas were located by light microscopy, and a laser with the excitation wavelength at 438 nm was used to focus and collect all Raman signals in the sample.

For the investigation on DNA damage, NR8383 cells were exposed to AG-QDs (0 and 200 μg/mL), H_2_O_2_ (50 μL/mL), or AG-QDs (200 μg/mL) with GSH (10 μg/mL) for 24 h at 37 °C. The DNA of NR8383 cells in each treatment was extracted using a Genomic DNA Mini Preparation Kit with a Spin Column (Beyotime Institute of Biotechnology, China) according to the manufacturer’s instructions. The extracted DNA was dissolved in DI water (20 ng/μL) and was deposited on a freshly cleaved mica substrate (1 cm × 1 cm). After washing and air-drying, the DNA chain morphology on the mica substrate was imaged by AFM in tapping mode. Moreover, the extracted DNA samples (20 ng/μL) from the un-exposed cells were mixed with AG-QDs (200 μg/mL), and then were allowed to sit for 24 h prior to morphological observation by AFM. In addition, ROS levels of AG-QDs alone and NR8383 cells after exposure to AG-QDs (200 μg/mL) were detected using 2,7-dichlorodihydrofluorescein diacetate (DCFH-DA, Beyotime Institute of Biotechnology, China) by a fluorescence spectrophotometer.

### Molecular docking simulation

To investigate the specific interaction between AG-QDs and DNA chains, ten representative structural models of AG-QDs (Additional file 1: Figure S18) based on the particle characterization data were employed. The binding mode for representative structural models of AG-QDs to DNA was investigated by CDOCKER, which was incorporated into Discovery Studio 2.5 (Accelrys Software Inc.) through the Dock Ligands protocol. CDOCKER is an implementation of the docking tool based on the CHARMm force field that has proven to be viable [57]. The crystal structure of DNA (PDB entry code: 1DJD) was retrieved from the Brookhaven Protein Database (PDB *http://www.rcsb.org/pdb*). Hydrogen atoms were added and the crystallographic waters were removed. The random DNA conformations were refined by grid-based simulated annealing in the receptor active site, which improved accuracy. From the above molecular docking simulation, insights into the specific interaction forces between the AG-QDs and DNA were obtained.

### RNA-seq analysis

After 24-h AG-QDs (100 μg/mL) exposure, NR8383 cells were sampled for RNA-Seq analysis through OE Biotech. Co., Ltd. (Shanghai, China). Briefly, the total RNA was extracted from the exposed NR8383 cells using a mirVana™ miRNA Isolation Kit (Thermo Scientific, USA). The integrity of the extracted RNA was evaluated using the Agilent 2100 Bioanalyzer (Agilent Technologies, USA). The cDNA, reverse-transcribed from the extracted RNA, was used to construct libraries by the TruSeq Stranded mRNA LTSample Prep Kit (Illumina, USA) according to the manufacturer’s protocol. cDNA libraries were sequenced on the Illumina sequencing platform (HiSeqTM 2500). Raw data (raw reads) generated after sequencing was processed using the NGS QC Toolkit and was then mapped to a reference *Rattus norvegicus* genome using Tophat. After comparing with the sequences of reference genes, the differentially expressed genes induced by AG-QDs were identified using the DESeq functions estimateSizeFactors and nbinomTest. A *P* value < 0.05 and Fold Change > 2 were set as the threshold for significantly differential expression.

### Statistical analysis

All experiments were run with triplicates or more, and the data were expressed with mean ± standard deviation. LSD and T tests were used to analyze the statistical significance using SPSS Statistics 20.0 (*p* < 0.05).

## Additional file

Additional file 1: **Figure S1.** Physicochemical characterization of AG-QDs. **Figure S2.** The hydrodynamic diameter of AG-QDs in DI water and cell culture medium. **Figure S3.** Cell viability of macrophages after exposure to AG-QDs at different concentrations. **Figure S4.** Viability of MC3T3-E1 cells after exposure to AG-QDs at different concentrations. **Figure S5.** Apoptosis of NR8383 cells during 24- and 48-h AG-QDs exposure. **Figure S6.** Export of AG-QDs by macrophages after AG-QDs internalization. **Figure S7.** Distribution of internalized AG-QDs in macrophages after exocytosis for 24 and 48 h. **Figure S8.** Confocal images of AG-QDs distributed in mitochondria, endo-lysosomes, and endoplasmic reticulum after incubation for 24 h. **Figure S9.** The effect of AG-QDs on the membrane stability of endo-lysosomes. **Figure S10.** Uptake of GO-QDs by NR8383 cells under confocal imaging. **Figure S11.** Confocal images of AG-QDs distributed in the cellular cytoplasm under 438 nm fluorescence emission. **Figure S12.** Distribution of AG-QDs in the cellular cytoplasm after incubation for 12 h. **Figure S13.** Relative expression of *Kapβ2* and *Nup98* after exposure to AG-QDs (200 μg/mL) for 12 and 24 h. **Figure S14.** The high content screening (HCS) images of NR8383 cells after AG-QDs (200 μg/mL) exposure in absence and presence of Hoechst33342 at 347 nm excitation and 483 nm emission. **Figure S15.** Raman images of cells after exposure to AG-QDs (200 μg/mL) for 24 and 48 h. **Figure S16.** ROS levels of AG-QDs (200 μg/mL) alone and NR8383 cells after AG-QDs exposure. **Figure S17.** AFM image of DNA chains that were directly exposed to AG-QDs-FBS (200 μg/mL) for 24 h. **Figure S18.** Ten representative structural models of AG-QDs. **Figure S19.** π-π Interactions between the AG-QDs (Structures 7–10) and DNA. **Table S1.** The number of π bonds between AG-QDs (Structures 1–10) and DNA as obtained by molecular docking. **Table S2.** The expression of genes in the caspase family after AG-QDs exposure. The macrophages were exposed to AG-QDs for 24 h prior to analysis. **Table S3.** Sequences of the gene-specific primers used in the quantitative real-time PCR (qRT-PCR) experiment. (DOCX 2467 kb)

## Abbreviations

AFM: atomic force microscopy
AG-QDs: aminated graphene quantum dots
CAD: caspase-activated DNase
CCK-8: Cell-Counting Kit 8
CLSM: confocal laser scanning microscopy
CLSM: confocal laser scanning microscopy
FBS: fetal bovine serum
GO: graphene oxide
HCS: High Content Screening
ICAD: inhibitor of caspase-activated DNase
*Kapβ2*: karyopherin *β*2
MIC: minimum inhibitory concentration
NPCs: nuclear pore complexes
NPs: nanoparticles
*Nup98*: nucleoporin 98
QDs: quantum dots
qRT-PCR: Reverse transcription quantitative polymerase chain reaction
rGO: reduced graphene oxide
RNA-Seq: RNA sequencing
ROS: reactive oxygen species
TEM: transmission electron microscopy
XPS: X-ray photoelectron spectroscopy

## References

[1] Trauzettel B, Bulaev DV, Loss D, Burkard G (2007). Spin qubits in graphene quantum dots. Nat Phys.

[2] Fatimy AE, Myers-Ward RL, Boyd AK, Daniels KM, Gaskill DK, Barbara P (2016). Epitaxial graphene quantum dots for high-performance terahertz bolometers. Nat Nanotechnol.

[3] Li Q, Chen B, Xing B (2017). Aggregation kinetics and self-assembly mechanisms of graphene quantum dots in aqueous solutions: cooperative effects of pH and electrolytes. Environ Sci Technol.

[4] Liu Q, Guo B, Rao Z, Zhang B, Gong J (2013). Strong two-photon-induced fluorescence from photostable, biocompatible nitrogen-doped graphene quantum dots for cellular and deep-tissue imaging. Nano Lett.

[5] Sun H, Gao N, Dong K, Ren J, Qu X (2014). Graphene quantum dots-band-aids used for wound disinfection. ACS Nano.

[6] Walkey C, Olsen J, Guo H, Emili A, Chan W (2012). Nanoparticle size and surface chemistry determine serum protein adsorption and macrophage uptake. J Am Chem Soc.

[7] Wu C, Wang C, Han T, Zhou X, Guo S, Zhang J (2013). Insight into the cellular internalization and cytotoxicity of graphene quantum dots. Adv Healthc Mater.

[8] Wang D, Zhu L, Chen J, Dai L (2015). Can graphene quantum dots cause DNA damage in cells?. Nanoscale.

[9] Yuan X, Liu Z, Guo Z, Ji Y, Jin M, Wang X (2014). Cellular distribution and cytotoxicity of graphene quantum dots with different functional groups. Nanoscale Res Lett.

[10] Zhu S, Zhang J, Tang S, Qiao C, Wang L, Wang H (2012). Surface chemistry routes to modulate the photoluminescence of graphene quantum dots: from fluorescence mechanism to up-conversion bioimaging applications. Adv Funct Mater.

[11] Gokhale R, Singh P (2014). Blue luminescent graphene quantum dots by photochemical stitching of small aromatic molecules: fluorescent nanoprobes in cellular imaging. Part Part Syst Charact.

[12] Qin Y, Zhou Z, Pan S, He Z, Zhang X, Qiu J (2015). Graphene quantum dots induce apoptosis, autophagy, and inflammatory response via p38 mitogen-activated protein kinase and nuclear factor-κB mediated signaling pathways in activated THP-1 macrophages. Toxicology.

[13] Vasconcelos D, Fonseca A, Costa M, Amaral I, Barbosa M, Águas A (2013). Macrophage polarization following chitosan implantation. Biomaterials.

[14] Lu L, Guo L, Wang X, Kang T, Cheng S (2016). Complexation and intercalation modes: a novel interaction of DNA and graphene quantum dots. RSC Adv.

[15] Zhou X, Zhang Y, Wang C, Wu X, Yang Y, Zheng B (2012). Photo-Fenton reaction of graphene oxide: a new strategy to prepare graphene quantum dots for DNA cleavage. ACS Nano.

[16] Liu B, Salgado S, Maheshwari V, Liu J (2016). DNA adsorbed on graphene and graphene oxide: fundamental interactions, desorption and applications. Curr Opin Colloid Interface Sci.

[17] Lu C, Huang PJ, Liu B, Ying Y, Liu J (2016). Comparison of graphene oxide and reduced graphene oxide for DNA adsorption and sensing. Langmuir.

[18] Junkermeier C, Solenov D, Reinecke T (2013). Adsorption of NH_2_ on graphene in the presence of defects and adsorbates. J Phys Chem C.

[19] Hu C, Liu Y, Yang Y, Cui J, Huang Z, Wang Y (2013). One-step preparation of nitrogen-doped graphene quantum dots from oxidized debris of graphene oxide. J Mater Chem B.

[20] Zhao J, Liu F, Wang Z, Cao X, Xing B (2015). Heteroaggregation of graphene oxide with minerals in aqueous phase. Environ Sci Technol..

[21] Huang S, Qiu H, Lu S, Zhu F, Xiao Q (2015). Study on the molecular interaction of graphene quantum dots with human serum albumin: combined spectroscopic and electrochemical approaches. J Hazard Mater.

[22] Rasel MAI, Li T, Nguyen TD, Singh S, Zhou Y, Xiao Y (2015). Biophysical response of living cells to boron nitride nanoparticles: uptake mechanism and bio-mechanical characterization. J Nanopart Res.

[23] Wang Z, Li N, Zhao J, White J, Qu P, Xing B (2012). CuO nanoparticle interaction with human epithelial cells: cellular uptake, location, export, and genotoxicity. Chem Res Toxicol.

[24] Sakhtianchi R, Minchin R, Lee K, Alkilany A, Serpooshan V, Mahmoudi M (2013). Exocytosis of nanoparticles from cells: role in cellular retention and toxicity. Adv Colloid Interf Sci.

[25] Chook YM, Süel KE (2011). Nuclear import by karyopherin-βs: recognition and inhibition. BBA-Mol Cell Res.

[26] Xu D, Farmer A, Chook YM (2010). Recognition of nuclear targeting signals by karyopherin-β proteins. Curr Opin Struct Biol.

[27] Franks TM, Hetzer MW (2013). The role of Nup98 in transcription regulation in healthy and diseased cells. Trends Cell Biol.

[28] De Gelder J, De Gussem K, Vandenabeele P, Moens L (2007). Reference database of Raman spectra of biological molecules. J Raman Spectrosc.

[29] Kang B, Austin L, El-Sayed M (2014). Observing real-time molecular event dynamics of apoptosis in living cancer cells using nuclear-targeted plasmonically enhanced raman nanoprobes. ACS Nano.

[30] Ren H, Wang C, Zhang J, Zhou X, Xu D, Zheng J (2010). DNA cleavage system of nanosized graphene oxide sheets and copper ions. ACS Nano.

[31] Ge X, Zhao X, Nakagawa A, Gong X, Skeen-Gaar R, Shi Y (2014). A novel mechanism underlies caspase-dependent conversion of the dicer ribonuclease into a deoxyribonuclease during apoptosis. Cell Res.

[32] Van Loo G, Schotte P, Van Gurp M, Demol H, Hoorelbeke B, Gevaert K (2001). Endonuclease g: a mitochondrial protein released in apoptosis and involved in caspase-independent DNA degradation. Cell Death Differ.

[33] Fink S, Cookson B (2006). Caspase1-dependent pore formation during pyroptosis leads to osmotic lysis of infected host macrophages. Cell Microbiol.

[34] Germain M, Affar E, D’Amours D, Dixit V, Salvesen G, Poirier G (1999). Cleavage of automodified poly (ADP-ribose) polymerase during apoptosis evidence for involvement of Caspase-7. J Biol Chem.

[35] Wolf B, Schuler M, Echeverri F, Green D (1999). Caspase-3 is the primary activator of apoptotic DNA fragmentation via DNA fragmentation factor-45/inhibitor of caspase-activated DNase inactivation. J Biol Chem.

[36] Sasidharan A, Panchakarla L, Sadanandan A, Ashokan A, Chandran P, Girish C (2012). Hemocompatibility and macrophage response of pristine and functionalized graphene. Small.

[37] Ma J, Liu R, Wang X, Liu Q, Chen Y, Valle R (2015). Crucial role of lateral size for graphene oxide in activating macrophages and stimulating pro-inflammatory responses in cells and animals. ACS Nano.

[38] Guo J, Liu D, Filpponen I, Johansson L, Malho J, Quraishi S (2017). Photoluminescent hybrids of cellulose nanocrystals and carbon quantum dots as cytocompatible probes for in vitro bio-imaging. Biomacromolecules.

[39] Hu X, Zhou Q (2013). Health and ecosystem risks of graphene. Chem Rev.

[40] Ouyang S, Hu X, Zhou Q (2015). Envelopment–internalization synergistic effects and metabolic mechanisms of graphene oxide on single-cell Chlorella vulgaris are dependent on the nanomaterial particle size. ACS Appl Mater Inter.

[41] Green P, Leeuwenburgh C (2002). Mitochondrial dysfunction is an early indicator of doxorubicin-induced apoptosis. Biochim Biophys Acta.

[42] Zhao F, Zhao Y, Liu Y, Chang X, Chen C, Zhao Y (2011). Cellular uptake, intracellular trafficking, and cytotoxicity of nanomaterials. Small.

[43] Zhang H, Zeng X, Li Q, Gaillard-Kelly M, Wagner C, Yee D (2009). Fluorescent tumour imaging of type I IGF receptor in vivo: comparison of antibody-conjugated quantum dots and small-molecule fluorophore. Br J Cancer.

[44] Martens TF, Remaut K, Demeester J, De Smedt SC, Braeckmans K (2014). Intracellular delivery of nanomaterials: how to catch endosomal escape in the act. Nano Today.

[45] Seo S, Chen M, Wang H, Kang M, Leong K, Kim H (2017). Extra-and intra-cellular fate of nanocarriers under dynamic interactions with biology. Nano Today.

[46] Hülsmann B, Labokha A, Görlich D (2012). The permeability of reconstituted nuclear pores provides direct evidence for the selective phase model. Cell.

[47] Jaworski S, Sawosz E, Kutwin M, Wierzbicki M, Hinzmann M, Grodzik M (2015). In vitro and in vivo effects of graphene oxide and reduced graphene oxide on glioblastoma. Int J Nanomedicine.

[48] Ziegler U, Groscurth P (2004). Morphological features of cell death. Physiology.

[49] Sangeetha Gowda KR, Mathew B, Sudhamani C, Naik H (2014). Mechanism of DNA binding and cleavage. J Biomed Biotechnol.

[50] Kim N, Kang J (2006). Oxidative damage of DNA induced by the cytochrome C and hydrogen peroxide system. J Biochem Mol Biol.

[51] Ding J, Gu Y, Li F, Zhang H, Qin W (2015). DNA nanostructure-based magnetic beads for potentiometric aptasensing. Anal Chem.

[52] Li F, Li X, Liu X, Zhang L, You L, Zhao J (2011). Noncovalent interactions between hydroxylated polycyclic aromatic hydrocarbon and DNA: molecular docking and QSAR study. Environ Toxicol Pha.

[53] Szabó C (1997). Ohshima H. DNA damage induced by peroxynitrite: subsequent biological effects. Nitric Oxide Biol Chem.

[54] Wang J, Wu Z, Zhang H, Li Y, Huang C (2017). Selective colorimetric analysis of spermine based on the cross-linking aggregation of gold nanoparticles chain assembly. Talanta.

[55] Ma L, Liu B, Huang P, Zhang X, Liu J (2016). DNA adsorption by ZnO nanoparticles near its solubility limit: implications for DNA fluorescence quenching and DNAzyme activity assays. Langmuir.

[56] Ghaemi M, Absalan G (2014). Study on the adsorption of DNA on Fe_3_O_4_ nanoparticles and on ionic liquid-modified Fe_3_O_4_ nanoparticles. Microchim Acta.

[57] Wu G, Robertson D, Brooks C, Vieth M (2003). Detailed analysis of grid-based molecular docking: a case study of CDOCKER - a CHARMm-based MD docking algorithm. J Comput Chem.

