# Supplementary material for: Graphene quantum dots in alveolar macrophage: uptake-exocytosis, accumulation in nuclei, nuclear responses and DNA cleavage
Source: Part Fibre Toxicol. 2018 Nov 13;15:45. doi: 10.1186/s12989-018-0279-8 (PMC6234698; doi:10.1186/s12989-018-0279-8)
Supplement: Supplementary file 1 — Figure S1. Physicochemical characterization of AG-QDs. Figure S2. The hydrodynamic diameter of AG-QDs in DI water and cell culture medium. Figure S3. Cell viability of macrophages after exposure to AG-QDs at different concentrations. Figure S4. Viability of MC3T3-E1 cells after exposure to AG-QDs at different concentrations. Figure S5. Apoptosis of NR8383 cells during 24- and 48-h AG-QDs exposure. Figure S6. Export of AG-QDs by macrophages after AG-QDs internalization. Figure S7. Distribution of internalized AG-QDs in macrophages after exocytosis for 24 and 48 h. Figure S8. Confocal images of AG-QDs distributed in mitochondria, endo-lysosomes, and endoplasmic reticulum after incubation for 24 h. Figure S9. The effect of AG-QDs on the membrane stability of endo-lysosomes. Figure S10. Uptake of GO-QDs by NR8383 cells under confocal imaging. Figure S11. Confocal images of AG-QDs distributed in the cellular cytoplasm under 438 nm fluorescence emission. Figure S12. Distribution of AG-QDs in the cellular cytoplasm after incubation for 12 h. Figure S13. Relative expression of Kapβ2 and Nup98 after exposure to AG-QDs (200 μg/mL) for 12 and 24 h. Figure S14. The high content screening (HCS) images of NR8383 cells after AG-QDs (200 μg/mL) exposure in absence and presence of Hoechst33342 at 347 nm excitation and 483 nm emission. Figure S15. Raman images of cells after exposure to AG-QDs (200 μg/mL) for 24 and 48 h. Figure S16. ROS levels of AG-QDs (200 μg/mL) alone and NR8383 cells after AG-QDs exposure. Figure S17. AFM image of DNA chains that were directly exposed to AG-QDs-FBS (200 μg/mL) for 24 h. Figure S18. Ten representative structural models of AG-QDs. Figure S19. π-π Interactions between the AG-QDs (Structures 7–10) and DNA. Table S1. The number of π bonds between AG-QDs (Structures 1–10) and DNA as obtained by molecular docking. Table S2. The expression of genes in the caspase family after AG-QDs exposure. The macrophages were exposed to AG-QDs for 24 h prior to anal [file 12989_2018_279_MOESM1_ESM.docx]

# Supporting Information

**Graphene quantum dots in alveolar macrophage: uptake-exocytosis, accumulation in nuclei, nuclear responses and DNA cleavage**

Lina Xu,^1,2^ Yanhui Dai,^2^ Zhenyu Wang,^1,3^ Jian Zhao,^2,3,^* Fei Li,^3,4^ Jason C. White,^5^ and Baoshan Xing^6^*

^1^ Institute of Environmental Processes and Pollution Control, and School of Environmental and Civil Engineering, Jiangnan University, Wuxi 214122, China

^2^ Institute of Coastal Environmental Pollution Control, and Ministry of Education Key Laboratory of Marine Environment and Ecology, Ocean University of China, Qingdao 266100, China

^3^ Laboratory for Marine Ecology and Environmental Science, Qingdao National Laboratory for Marine Science and Technology, Qingdao 266071, China

^4^ Key Laboratory of Coastal Zone Environmental Processes and Ecological Remediation, Yantai Institute of Coastal Zone Research (YIC), Chinese Academy of Sciences (CAS), Yantai 264003, China

^5^ Department of Analytical Chemistry, The Connecticut Agricultural Experiment Station, New Haven, Connecticut 06504, United States

^6^ Stockbridge School of Agriculture, University of Massachusetts, Amherst, Massachusetts 01003, United States

*Corresponding authors

*E-mail address:* [bx@umass.edu](mailto:bx@umass.edu) (Dr. Baoshan Xing); [jzhao@ouc.edu.cn](mailto:jzhao@ouc.edu.cn) (Dr. Jian Zhao)

**a**

**b**

|  | Elemental  Content% |  | Atomic ratio |
| --- | --- | --- | --- |
| C | 54.33 | O/C | 0.525 |
| O | 38.05 | N/C | 0.140 |
| N | 7.62 | O+N/C | 0.665 |

C

**43.6%**

**11.8%**

**12.3%**

**13.6%**

**18.7%**

N-H

-N^+^=

-N=C

**38.1%**

**9.1%**

**52.8%**

**c**

C

Figure S1. Physicochemical characterization of AG-QDs. (**a**) Elemental analysis of AG-QDs using XPS. (**b**) The C 1s XPS spectra of AG-QDs. (**c**) N 1s XPS spectra of the AG-QDs.

AG-QDs

in DI water

AG-QDs

in medium

Figure S2. The hydrodynamic diameter of AG-QDs (50 μg/mL) in DI water and cell culture medium. The values are given as the mean ± SD (standard deviation).

d

e

c

bc

b

a

a

b

e

d

c

b

b

a

a

b

a

a

a

a

a

c

c

b

a

a

a

a

a

a

a

a

a

a

a

Figure S3. Cell viability of macrophages after exposure to AG-QDs at different concentrations (0, 10, 25, 50, 100, 200, and 500 μg/mL). Cell viability was determined using CCK-8 assay after AG-QDs exposure for 12, 24, 48, 72 and 96 h. The values are given as the mean ± SD (standard deviation). For a given exposure time, significant differences among exposure concentrations of AG-QDs (0-500 μg/mL) are marked with different letters (*p* < 0.05, LSD test, n=6).

Figure S4. Viability of MC3T3-E1 cells after exposure to AG-QDs at different concentrations (0, 10, 25, 50, 100, 200, and 500 μg/mL). Cell viability was determined using CCK-8 assay after AG-QDs exposure for 12, 24, 48, 72 and 96 h. The values are given as the mean ± SD (standard deviation). For a given exposure time, significant differences among exposure concentrations of AG-QDs (0-500 μg/mL) are marked with different letters (*p* < 0.05, LSD test, n=6).

a

b

a

a

ab

b

c

b

c

c

bc

a

a

a

b

a

Figure S5. Apoptosis of NR8383 cells during 24- (**a**) and 48-h (**b**) AG-QDs exposure. The values are given as the mean ± SD (standard deviation). For early apoptosis or late apoptosis, significant difference among AG-QDs concentrations is marked with different letters (*p* < 0.05, LSD test, n=6).

a


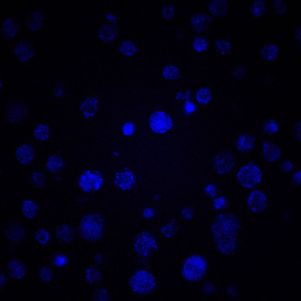

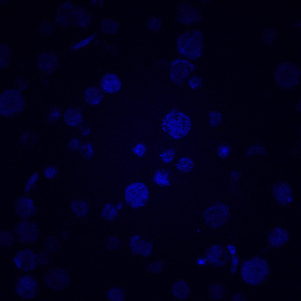

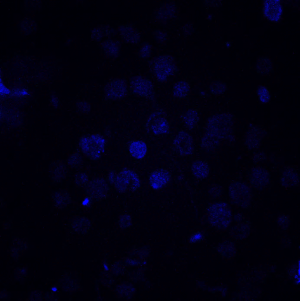


Exocytosis for 24 h

ab

ab

ab

ab

ab

de

cd

bc

bc

ab

cde

de

cd

e

a

f

ef

b

a

b

a

b

Exocytosis for 48 h

Before Exocytosis

15 μm

Figure S6. Export of AG-QDs by macrophages after AG-QDs internalization. (**a**) Macrophages were incubated with AG-QDs (50, 100 and 200 μg/mL) for 24 h, then washed, and incubated with fresh cell medium without AG-QDs. The fluorescence intensity of cells in the amended medium was determined after incubation for 2, 4, 6, 8, 12, 24 and 48 h. The values are given as the mean ± SD (standard deviation). For a given pre-exposure concentration, significant difference among different exocytosis times is marked with different letters (*p* < 0.05, LSD test, n=6). (**b**) Confocal images of macrophages before exocytosis (incubated with 200 μg/mL AG-QDs for 24 h), and after exocytosis for 24 and 48 h.

a

Overlay

SYTO 9

AG-QDs

Bright field


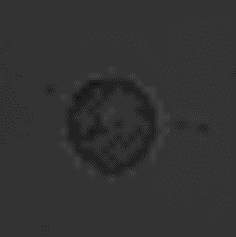

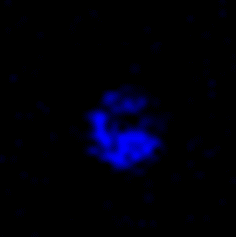

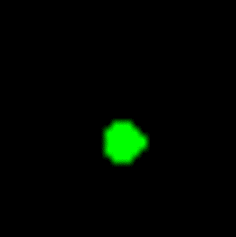

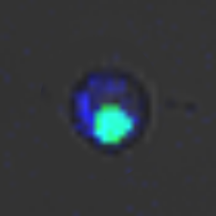


b

Nucleus

s

Nucleus

s

5 μm


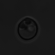

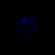

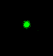

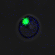


Nucleus

s

Nucleus

s

Figure S7. Distribution of internalized AG-QDs in cellular nucleus after exocytosis for 24 (**a**) and 48 h (**b**). Macrophages were incubated with AG-QDs (200 μg/mL) for 24 h, then washed, and incubated with fresh cell medium without AG-QDs. After re-incubation for 24 and 48 h, the nuclei were stained by SYTO 9 for 1 h, and the confocal images were captured. It was shown that the internalized AG-QDs remained in the nucleus (red arrow) ever after 48-h excretion.

Nucleus

Overlay

AG-QDs

Bright field


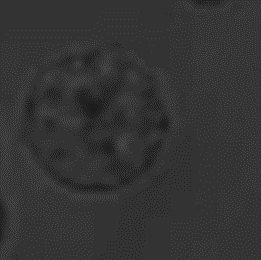

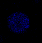

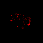

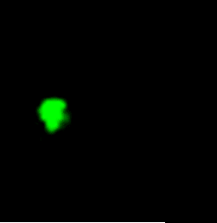

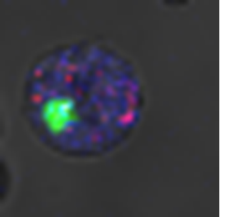


Mitochondria

5 μm


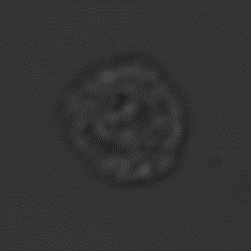

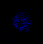

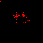

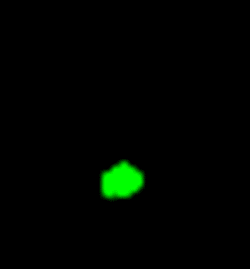

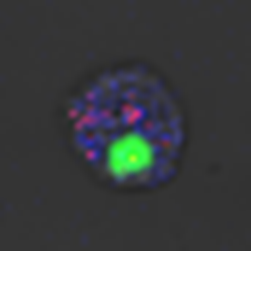


Endoplasmic

reticulum

Endo-Lysosomes


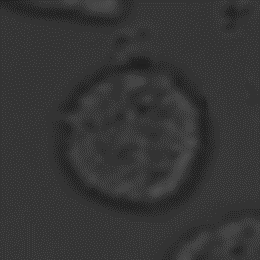

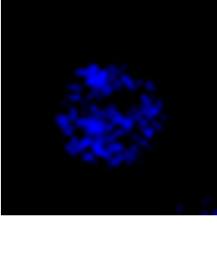

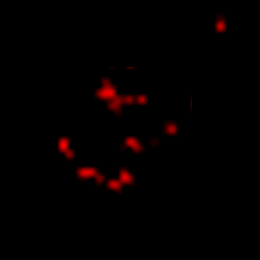

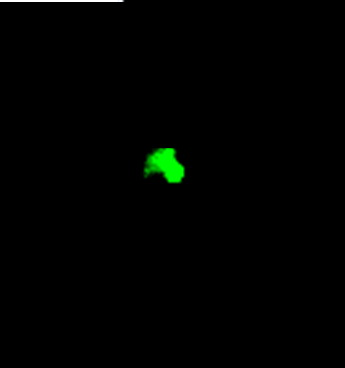

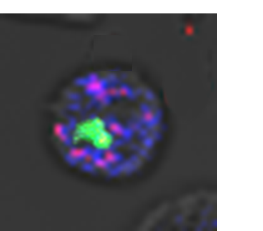


Figure S8. Confocal images of AG-QDs (200 μg/mL) distributed in mitochondria, endo-lysosomes, and endoplasmic reticulum after incubation for 24 h. Mitochondria, endo-lysosomes, endoplasmic reticulum and nuclei were stained by Mito-Tracker Red, Lyso-Tracker Red, ER-Tracker Red, and SYTO 9 (Green), respectively.

a

Lyso-Tracker Red

Bright field


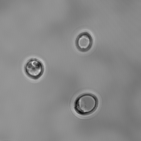

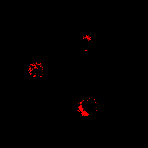


10 μm

c

b


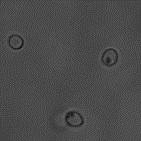

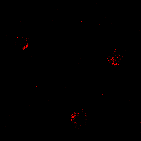


Figure S9. The effect of AG-QDs on the membrane stability of endo-lysosomes. (**a**) and (**b**): Confocal images of endo-lysosomes (stained by Lyso-Tracker Red) in NR8383 cells after exposure to AG-QDs (200 μg/mL) for 0 and 24 h, respectively. (**c**): The fluorescence intensities of endo-lysosomes. In panel **c**, significant difference between the fluorescence intensities of normal cells (CK) and treated cells is marked with “*” (*p* < 0.05, T test, n=3).

| Material | Surface functional groups (%) ^a^ | | | | Elemental  Content (%) ^a^ | | O/C atomic ratio ^a^ |
| --- | --- | --- | --- | --- | --- | --- | --- |
|  | C=C, C-C | C-OH | C=O | O=C-OH | C | O |  |
| GO-QDs | 58.1 | 18.3 | 18.8 | 3.6 | 61.86 | 38.14 | 0.463 |

b

a

| Material | Size (nm)^b^ | Thickness (nm)^c^ | Zeta potential  (mv)^d^ | Hydrodynamic diameter (nm)^d^ |
| --- | --- | --- | --- | --- |
| GO-QDs in DI water | 7.84±0.24 | 1.51±0.06 | -37.4±2.67 | 34.8±3.65 |
| GO-QDs in culture medium | 19.4±1.02 | 7.51±0.27 | -10.9±1.49 | 150.4±18.6 |

^a^ Observed from XPS;

^b^ Size observed from TEM micrographs;

^c^ Thickness observed from AFM micrographs;

^d^ Zeta potential and hydrodynamic diameter observed from Zetasizer.


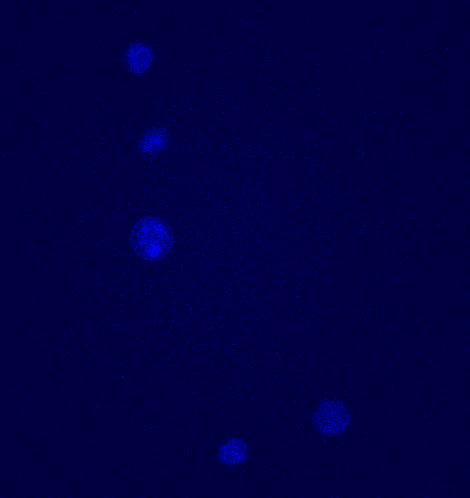

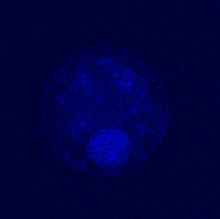

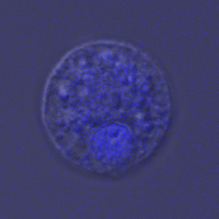


e

c

3 μm

3 μm

10 μm

Nucleus

Nucleus

d

Figure S10. (**a**) and (**b**): Characterization of GO-QDs (i.e., the two small tables at the top). (**c**): Uptake of GO-QDs (200 μg/mL) by NR8383 cells under confocal imaging. (**d**): The enlarged image from the red box of panel (**c**). (**e**): The merged fluorescence and bright field image of panel (**d**). After exposure to GO-QDs (200 μg/mL) for 24 h, GO-QDs entered cells and accumulated in nucleus.


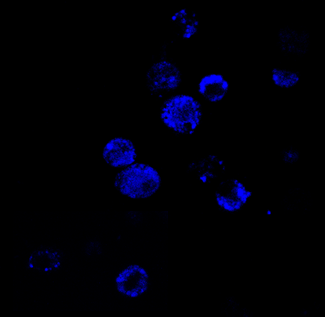

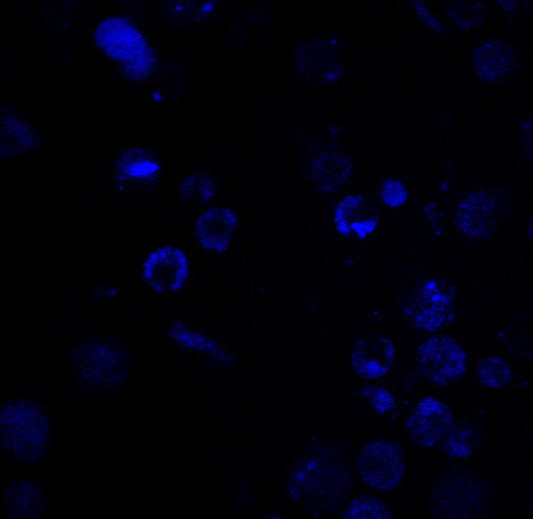

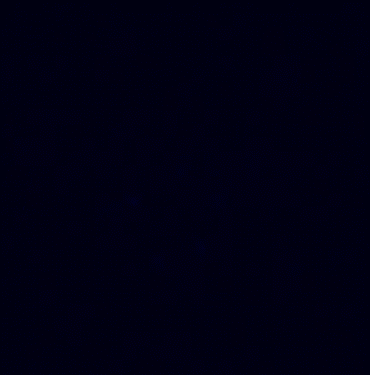


200 μg/mL

100 μg/mL

0 μg/mL

15 μm

Figure S11. Confocal images of AG-QDs distributed in the cellular cytoplasm under 438 nm fluorescence emission (blue). After incubation with AG-QDs (100 or 200 μg/mL) for 12 h, AG-QDs were mainly aggregated in cellular cytoplasm, rather than nucleus (red arrows).

Bright field

AG-QDs

SYTO 9

Overlay


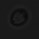

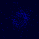

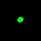

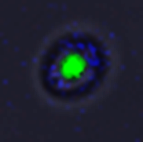


5 μm

Nucleus

s

Nucleus

s

Figure S12. Distribution of AG-QDs (200 μg/mL) in the cellular cytoplasm after incubation for 12 h. After 12-h exposure, the cells were washed and the nuclei were stained by SYTO 9 for 1 h. The confocal images showed that AG-QDs were mainly aggregated in cellular cytoplasm, rather than nucleus (red arrow).

b

ab

a

b

b

a

Figure S13. Relative expression of *Kapβ2* and *Nup98* after exposure to AG-QDs (200 μg/mL) for 12 and 24 h. The values were given as the mean ± SD (standard deviation). For a given gene, significant difference among the exposure times is marked with different letters (*p* < 0.05, LSD, n=3).


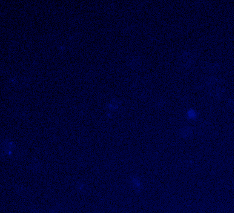

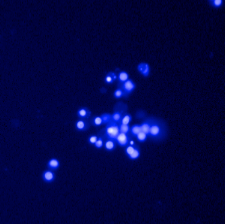


20 μm

AG-QDs + Hoechst

AG-QDs

AG-QDs + Hoechst

AG-QDs

b

a

Figure S14. The high content screening (HCS) images of NR8383 cells after AG-QDs (200 μg/mL) exposure in the absence (**a**) and presence (**b**) of Hoechst33342 at 347 nm excitation and 483 nm emission. (**c**): Fluorescence intensities of NR8383 cells that were quantified from panel (a) and (b). It is shown that fluorescence intensity in cells exposed to AG-QDs was only 8.93% of that in AG-QDs-exposed cell after staining by Hoechst33342. Thereby, the fluorescence intensity of AG-QDs at 347 nm excitation and 483 nm emission can be ignored.

a


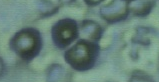


Protein C=N

Phenyla-

lanine

PO_2_^-^stretching vibration

AG-QDs 24 h

d

b

Normal cell


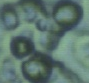


DNA framework,

dT, dC

lipids C=O

AG-QDs 48 h

c


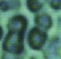


D

G

Figure S15. (**a-c**) Raman images of cells after exposure to AG-QDs (200 μg/mL) for 24 and 48 h. The nucleus areas that are marked with red squares were investigated using Raman spectra (**d**).

AG-QDs

a

b

Culture medium

Treated cells

Normal cells

Figure S16. ROS levels of AG-QDs (200 μg/mL) alone (**a**) and NR8383 cells after AG-QDs (200 μg/mL) exposure for 24 h (**b**). ROS generation was detected using 2,7-dichlorodihydrofluorescein diacetate (DCFH-DA, Beyotime Institute of Biotechnology, China), which is an oxidation-sensitive fluorescent probe dye. The values are given as the mean ± SD (standard deviation). In panel **a,** no significant difference between culture medium and AG-QDs groups was observed. In panel **b**, significant difference between the ROS levels of normal cells and AG-QDs-treated cells is marked with “*” (*p* < 0.05, T test, n=3).


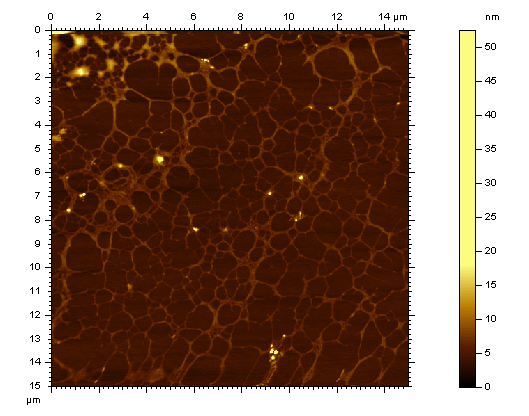


Figure S17. AFM image of DNA chains that were directly exposed to AG-QDs-FBS (200 μg/mL) for 24 h. The inserted figure was the height profiles of DNA chains. White arrows indicate the particles on DNA chains.

| 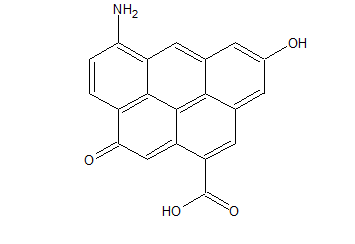 | 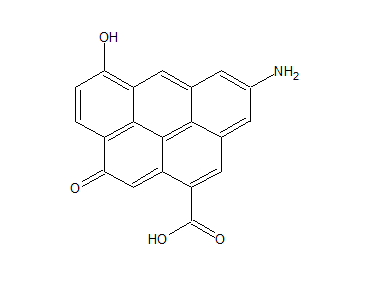 |
| --- | --- |
| Structure 1 | Structure 2 |
| 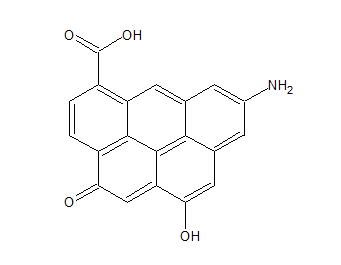 | 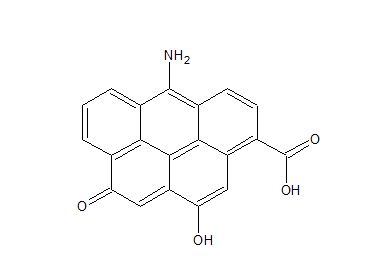 |
| Structure 3 | Structure 4 |
| 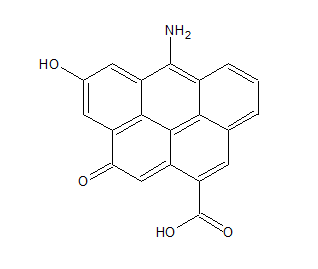 | 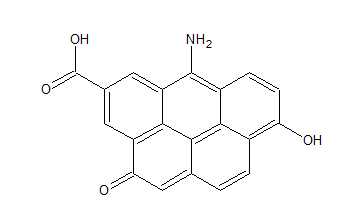 |
| Structure 5 | Structure 6 |
| 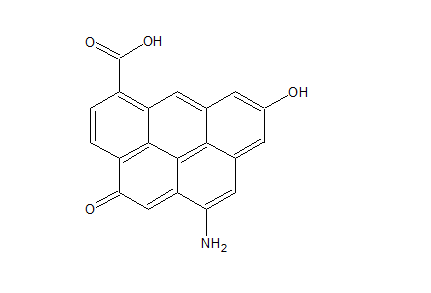 | 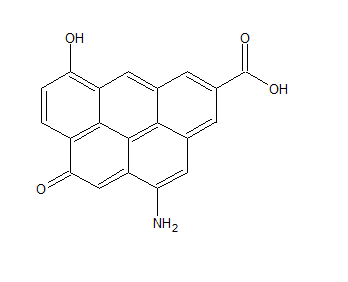 |
| Structure 7 | Structure 8 |
| 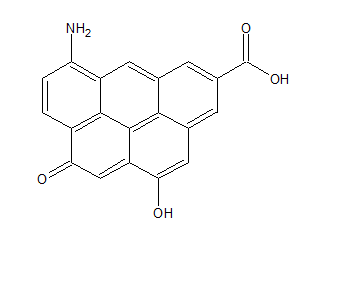 | 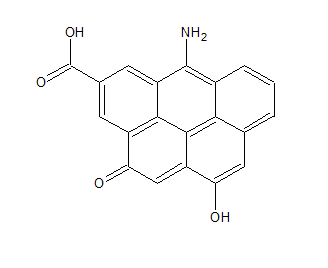 |
| Structure 9 | Structure 10 |

Figure S18. Ten representative structural models of AG-QDs. The functional groups on these AG-QDs models were obtained from XPS analysis.

| **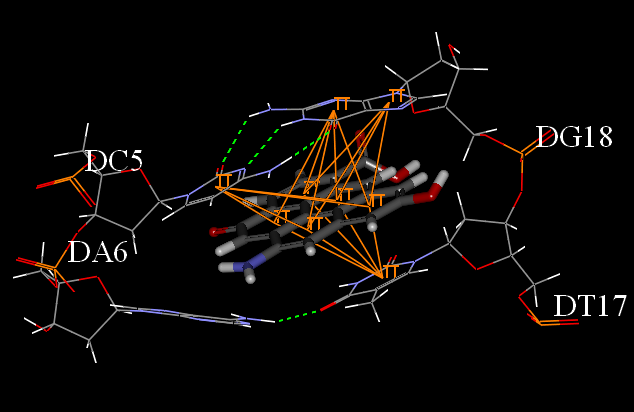** | 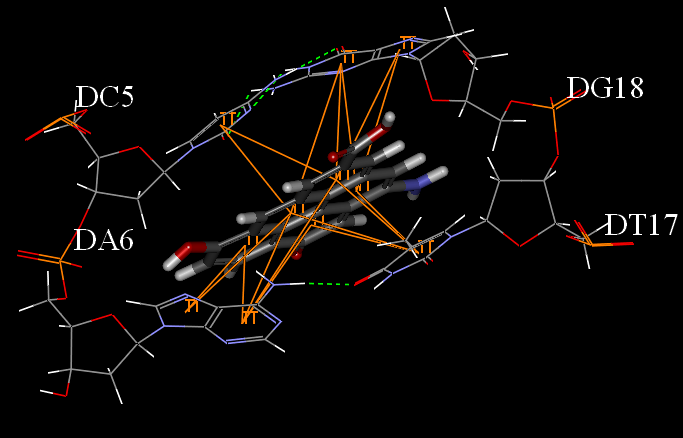 |
| --- | --- |
| Structure 7 | Structure 8 |
| 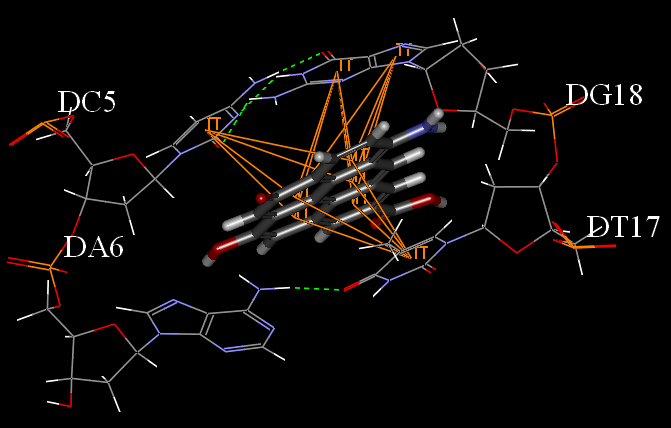 | 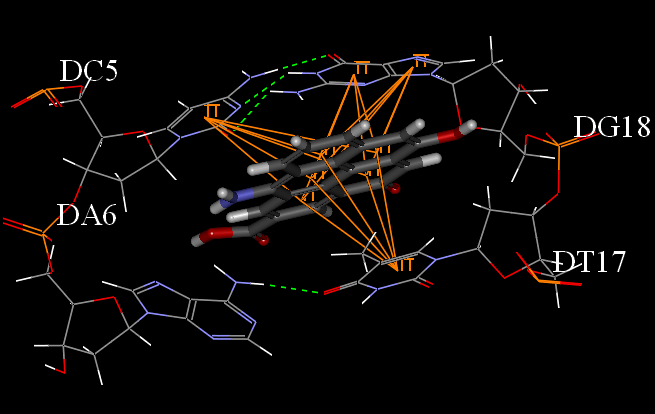 |
| Structure 9 | Structure 10 |

Figure S19. π-π Interactions between the AG-QDs (Structures 7-10) and DNA. Yellow lines show π-π interactions between AG-QDs and DNA base groups. Green dotted lines show H-bonds between purines and pyrimidines. Carbon is colored in grey, oxygen in red, hydrogen in white, and nitrogen in blue. For Structures 7-10, only π-π interactions with DNA were detected via molecular docking simulation.

Table S1. The number of π bonds between AG-QDs (Structures 1-10) and DNA chains as obtained by molecular docking.

| Structure | 1 | 2 | 3 | 4 | 5 | 6 | 7 | 8 | 9 | 10 |
| --- | --- | --- | --- | --- | --- | --- | --- | --- | --- | --- |
| Numbers of π bonds | 17 | 17 | 18 | 16 | 11 | 18 | 18 | 13 | 17 | 18 |

Table S2. The expression of genes in the caspase family after AG-QDs exposure. The macrophages were exposed to AG-QDs for 24 h prior to analysis.

| RGD ID | Symbol | Gene name | Description | Fold change | *P* value |
| --- | --- | --- | --- | --- | --- |
| 731729 | *Casp1* | *Caspase-1* | encodes a protein that exhibits cysteine-type endopeptidase activity | 2.709 | 6.33E^-71^ |
| 731460 | *Casp2* | *Caspase-2* | encodes the evolutionarily ancient and most conserved member of the cysteine proteases that plays an important role in stress-induced apoptosis, DNA repair and tumor suppression | ND | ND |
| 10289 | *Casp3* | *Caspase-3* | function as essential regulators of programmed cell death through apoptosis. | ND | ND |
| 1553228 | *Casp4* | *Caspase-4* | apoptosis-related cysteine peptidase, encodes a member of the cysteine proteases that plays important roles in apoptosis, cell migration and inflammatory response. | 3.0902 | 2.67E^-84^ |
| 732007 | *Casp6* | *Caspase-6* | encodes a member of the cysteine proteases that plays important roles in regulating apoptosis and neurodegeneration. | ND | ND |
| 1553182 | *Casp7* | *Caspase-7* | encodes a protein that exhibits aspartic-type endopeptidase activity; cysteine-type endopeptidase activity; cysteine-type endopeptidase activity involved in apoptotic process | 3.3177 | 2.10E^-54^ |
| 730847 | *Casp8* | *Caspase-8* | an initiator of apoptotic cell death | 10.684 | 0 |
| 62159 | *Casp9* | *Caspase-9* | well studied for its involvement in immune and apoptosis signaling; the initiator caspase, is activated after cytochrome c release from mitochondria | ND | ND |
| 731442 | *Casp12* | *Caspase-12* | encodes a protein that exhibits cysteine-type endopeptidase activity | ND | ND |
| 1323415 | *Casp14* | *Caspase-14* | encodes a protein that exhibits cysteine-type endopeptidase activity (inferred) | ND | ND |

Note: RGD: Rat Genome Database; ND: Differential expression of this gene was not detected.

Table S3. Sequences of the gene-specific primers used in the quantitative real-time PCR (qRT-PCR) experiment.

| Gene name | Forward Primer Sequence | Reverse Primer Sequence |
| --- | --- | --- |
| 18S rRNA | 5'-GTTGCAGTTAAAAAGCTCGT-3' | 5'-TTGATTTCTCATAAGGTGCC-3' |
| *Kapβ2* | 5'- TTCGAATGGATCGCCTGCTT-3' | 5'- CCGTCCTCGATCGGTGAAAA-3' |
| *Nup98* | 5'- CAGGCACAGCCAAATCACAT -3' | 5'- CCGTTGGCTAGAGATGGTTCA -3' |
